# *Ziziphus jujuba* Mill. Suspension Ameliorates Scopolamine‐Induced Cognitive Impairment via PTGS2‐Centered Neuroinflammatory Signaling

**DOI:** 10.1155/mi/8871660

**Published:** 2026-05-07

**Authors:** Qi Liu, Yihan Wang, Binchuan Wang, Jinyu Wang, Mingwei Zhang, Yi Liu, Xuefeng Min

**Affiliations:** ^1^ Department of Neurosurgery, The Affiliated Traditional Chinese Medicine Hospital, Southwest Medical University, Luzhou, 646000, China, swmu.edu.cn; ^2^ Department of Nephrology, The Affiliated Traditional Chinese Medicine Hospital, Southwest Medical University, Luzhou, 646000, China, swmu.edu.cn

**Keywords:** cognitive impairment, medicinal foods, network pharmacology, neuroinflammation, PTGS2, scopolamine, *Ziziphus jujuba*

## Abstract

**Background:**

Cognitive impairment is a common feature of neurodegenerative diseases, in which sustained neuroinflammation critically contributes to neuronal dysfunction and memory decline. As a representative condition, Alzheimer’s disease (AD) provides key insights into inflammation‐associated cognitive impairment. However, current anti‐inflammatory interventions exhibit limited efficacy and potential adverse effects, highlighting the need for safer strategies targeting neuroinflammation‐related cognitive dysfunction.

**Objective:**

Guided by the concept of food–medicine homology, this study aimed to elucidate the molecular mechanisms by which *Ziziphus jujuba* alleviates cognitive impairment associated with neuroinflammation.

**Methods:**

AD‐relevant targets associated with cognitive dysfunction were obtained from Gene Expression Omnibus (GEO) and GeneCards, and active compounds of *Z. jujuba* were retrieved from traditional Chinese medicine systems pharmacology (TCMSP). Shared targets were prioritized using least absolute shrinkage and selection operator (LASSO), random forest (RF), and support vector machine (SVM) algorithms. The diagnostic value of the core target was evaluated by receiver operating characteristic (ROC) analysis and a nomogram model with calibration and decision curve analysis (DCA). Functional enrichment, localization analyses, and molecular docking were performed. Experimental validation was conducted in a scopolamine‐induced cognitive impairment mouse model using the Morris water maze (MWM), histopathology, and western blotting.

**Results:**

PTGS2 was identified as a key inflammatory target associated with neuroinflammation‐related cognitive impairment and was enriched in the NOD‐like receptor (NLR) signaling pathway. ROC and nomogram analyses indicated good diagnostic and predictive performance (area under the curve [AUC] > 0.7). PTGS2 was localized on chromosome 1 and showed relatively high expression in the cerebral cortex. *Z. jujuba* compounds exhibited strong binding to PTGS2 (ΔG ≤ −8.0 kcal/mol). In vivo, *Z. jujuba* improved cognitive performance, alleviated hippocampal injury, and downregulated PTGS2 and related inflammatory signaling pathways, including NLRP3/NF‐κB/MAPK and interleukin (IL)‐1β.

**Conclusion:**

This study demonstrates that *Z. jujuba* ameliorates neuroinflammation‐related cognitive dysfunction primarily by suppressing PTGS2‐centered inflammatory signaling. Integrating computational analyses and in vivo validation in scopolamine‐induced mice, our findings support *Z. jujuba* as a safe multitarget intervention for inflammation‐associated cognitive impairment and highlight the potential of food–medicine homology in neuroinflammatory cognitive disorders.

## 1. Introduction

With the rapid aging of the global population, the prevalence of Alzheimer’s disease (AD) continues to rise, with projections estimating approximately 139 million affected individuals worldwide by 2050 [1]. Cognitive impairment, a core and debilitating clinical manifestation of AD, severely compromises patients’ quality of life and imposes a substantial burden on families and healthcare systems [2]. The pathogenesis of AD is highly complex, involving multiple interrelated mechanisms, including amyloid‐β (Aβ) plaque deposition, tau hyperphosphorylation and neurofibrillary tangle formation, synaptic dysfunction, and progressive neuronal loss [3]. Among these processes, accumulating evidence has identified chronic neuroinflammation as a critical driver of disease progression rather than a mere secondary consequence [4, 5]. Sustained neuroinflammation, predominantly mediated by aberrant microglial activation, plays a central role in amplifying neuronal injury and accelerating cognitive decline, particularly in neurodegenerative conditions such as AD [6]. Excessive release of pro‐inflammatory cytokines and chemokines disrupts neuronal homeostasis, impairs synaptic plasticity, and promotes neurodegeneration. Importantly, neuroinflammation forms a self‐perpetuating feed‐forward loop with classical pathological features observed in AD [7]: inflammatory signaling enhances amyloidogenic processing and Aβ deposition, while simultaneously facilitating tau hyperphosphorylation via inflammation‐associated kinases, thereby exacerbating synaptic dysfunction and cognitive impairment. Collectively, this intertwined inflammatory network highlights neuroinflammation as a rational and promising therapeutic target for mitigating neuroinflammation‐associated cognitive decline, including that observed in neurodegenerative conditions.

Although nonsteroidal anti‐inflammatory drugs (NSAIDs) have been extensively investigated as potential modulators of neuroinflammation in AD, multiple large‐scale phase III clinical trials have failed to demonstrate meaningful cognitive benefits. Long‐term NSAID administration is also associated with significant gastrointestinal and systemic adverse effects, limiting their suitability for chronic use in elderly populations [8]. Moreover, the limited efficacy of NSAIDs in AD is increasingly attributed to their narrow pharmacological focus—primarily targeting cyclooxygenase (COX) activity—which fails to adequately address the complex, multinode inflammatory networks underlying neurodegeneration. In some cases, nonselective COX inhibition may even exacerbate hippocampal atrophy or fail to significantly alter Aβ or tau pathology [9]. These limitations underscore the urgent need for safer, multitarget therapeutic strategies capable of modulating neuroinflammation‐driven cognitive dysfunction.

*Ziziphus jujuba* (jujube), a classic “food–medicine homology” substance in traditional Chinese medicine (TCM), has long been used to nourish qi, soothe the mind, and support cognitive vitality. It also possesses a well‐documented safety profile for long‐term oral administration—an attribute particularly valuable for elderly populations requiring chronic intervention [10–12]. While its traditional applications primarily provide a historical rationale for modern investigation, growing pharmacological evidence highlights the relevance of *Z. jujuba* to neuroinflammation‐associated cognitive dysfunction. *Z. jujuba* contains a diverse spectrum of bioactive constituents, including flavonoids (such as quercetin and rutin), polysaccharides, jujubosides, and triterpenoids [13–15]. Among these, flavonoids are widely recognized for their potent anti‐inflammatory and antioxidant activities in neuroinflammatory contexts; polysaccharides modulate immune responses by regulating microglial activation states; jujubosides exert neuroprotective effects through inhibition of pro‐inflammatory signaling; and triterpenoids contribute to alleviating neuroinflammation‐induced neuronal damage. Collectively, these constituents exert synergistic and multitarget regulatory effects on neuroinflammatory processes. Importantly, *Z. jujuba*–derived components have been reported to modulate key pathological nodes of neuroinflammation, including aberrant microglial activation, excessive release of pro‐inflammatory cytokines (e.g., interleukin [IL]‐1β and tumor necrosis factor [TNF]‐α), and activation of inflammation‐related signaling pathways (NF‐κB and MAPK) that are closely associated with neurodegenerative processes [16–18]. Concurrently, these bioactive constituents attenuate inflammation‐induced oxidative stress by scavenging reactive oxygen species (ROS) and enhancing endogenous antioxidant defenses, such as superoxide dismutase and glutathione peroxidase, thereby potentially disrupting the self‐perpetuating feed‐forward loop between neuroinflammation and neurodegenerative pathology that contributes to cognitive decline [19, 20].

Collectively, this multinode regulatory capacity, coupled with its favorable safety profile, distinguishes *Z. jujuba* from conventional nonselective anti‐inflammatory agents that target limited pathways and carry systemic adverse effects. From a translational perspective, the multicomponent and multitarget nature of *Z. jujuba* is well‐suited to the complex pathophysiology of neurodegenerative disorders, in which neuroinflammation intersects with multiple degenerative cascades. Accordingly, network pharmacology provides an effective framework for elucidating how the bioactive constituents of *Z. jujuba* interact with disease‐associated molecular networks, while machine learning approaches further refine target prioritization and pathway relevance [21]. Importantly, given that the primary aim of this study was to investigate neuroinflammation‐associated cognitive dysfunction rather than to directly model, validate, or infer AD‐specific mechanisms. In this study, we integrated network pharmacology, machine learning, molecular docking, and experimental validation to systematically investigate the molecular mechanisms underlying the therapeutic effects of *Z. jujuba* on neuroinflammation‐related cognitive dysfunction. To experimentally evaluate the predicted mechanisms within the context of neuroinflammation‐associated cognitive impairment, a scopolamine‐induced mouse model was employed.

## 2. Materials and Methods

### 2.1. Research Design

**Figure 1:**
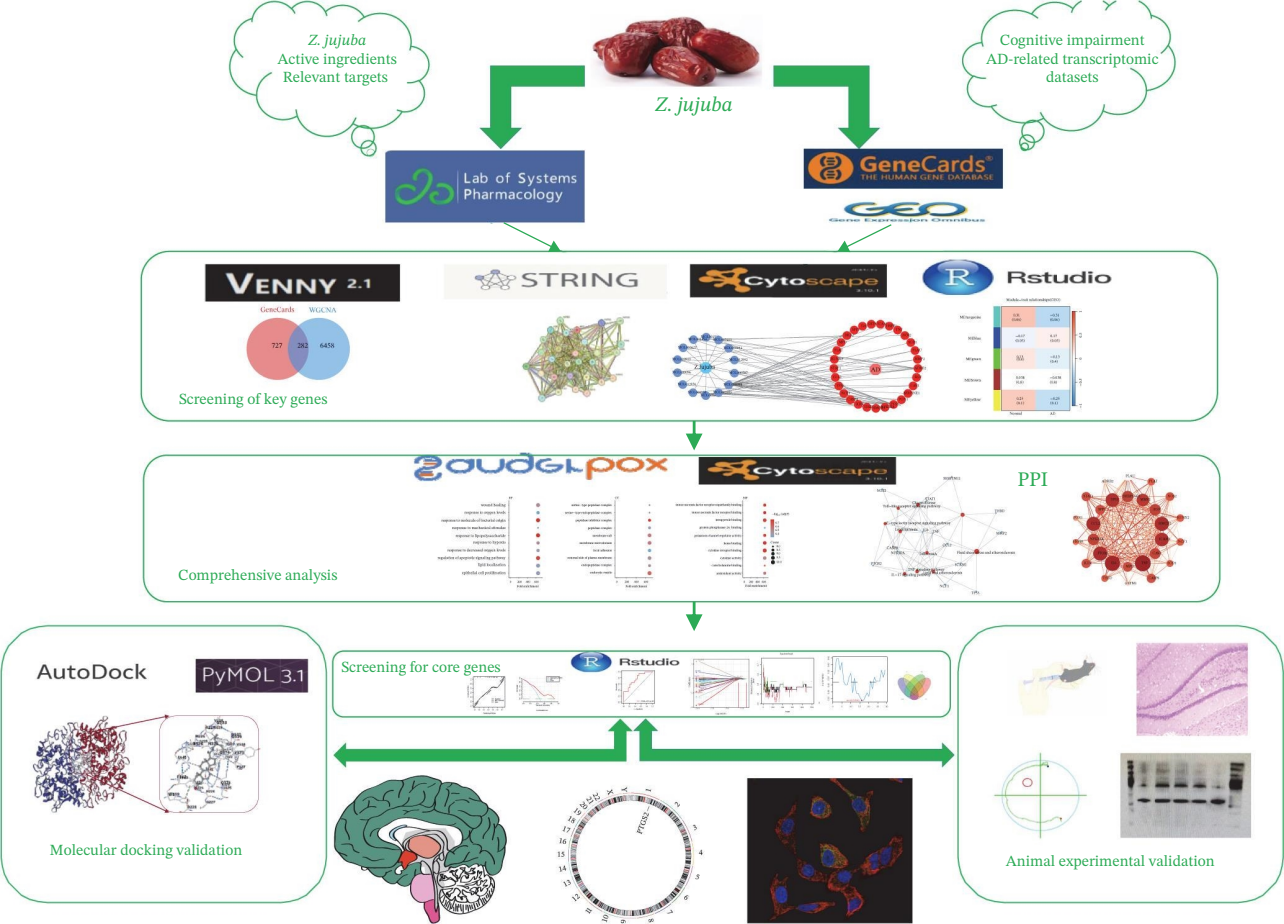
Flowchart illustrates an integrated analytical strategy for elucidating the mechanisms by which *Z. jujuba* ameliorates neuroinflammation‐associated cognitive impairment through modulation of neuroinflammatory signaling. AD‐related transcriptomic datasets were utilized as representative sources of neurodegeneration‐associated gene expression patterns.

As illustrated in Figure 1, this study employed an integrative strategy combining network pharmacology, machine learning, molecular docking, and experimental validation to elucidate the therapeutic mechanisms of *Z. jujuba* in neuroinflammation‐associated cognitive impairment. Publicly available AD‐related transcriptomic datasets were used as a representative source of neurodegeneration‐associated gene expression patterns to facilitate the identification of molecular targets potentially involved in inflammation‐driven cognitive dysfunction, rather than to directly model AD‐specific pathological processes.

First, disease‐related gene expression profiles were retrieved from the Gene Expression Omnibus (GEO) database, while disease‐associated targets were collected from the GeneCards database to characterize molecular networks associated with neurodegeneration and cognitive impairment. In parallel, the active constituents of *Z. jujuba* and their corresponding putative targets were obtained from the TCM systems pharmacology (TCMSP) database. The intersecting targets between disease‐related genes and *Z. jujuba* targets were identified as potential regulatory targets relevant to cognitive dysfunction. These common targets were imported into the STRING database to construct a protein–protein interaction (PPI) network, which was visualized and topologically analyzed using Cytoscape software to identify hub genes. Gene Ontology (GO) functional annotation and Kyoto Encyclopedia of Genes and Genomes (KEGG) pathway enrichment analyses were subsequently performed to elucidate the biological functions and signaling pathways associated with these targets. To further improve target prioritization accuracy, machine learning algorithms were applied to screen key targets. Receiver operating characteristic (ROC) curve analysis and diagnostic nomogram modeling were subsequently conducted to evaluate the potential predictive performance of these candidate genes. Gene set enrichment analysis (GSEA) was then performed to explore pathway‐level alterations associated with neuroinflammatory signaling and cognitive dysfunction. Molecular docking analysis was conducted to assess the binding affinity between major bioactive compounds of *Z. jujuba* and the identified core targets.

To further assess the robustness and biological relevance of the screened targets, an independent transcriptomic dataset derived from human hippocampal tissue was introduced as an external validation cohort, as the hippocampus plays a central role in learning and memory processes and is highly relevant to cognitive function. Finally, a scopolamine‐induced mouse model of cognitive impairment was established for experimental validation. Cognitive function was evaluated using the Morris water maze (MWM) test, hippocampal histopathological alterations were examined by hematoxylin–eosin (HE) staining, and the expression of downstream signaling molecules associated with the identified core targets was analyzed using western blotting. These experiments were designed to validate the potential molecular mechanisms by which *Z. jujuba* ameliorates neuroinflammation‐associated cognitive dysfunction, with the in vivo model reflecting functional modulation of cognition and neuroinflammation rather than AD‐specific pathological processes such as Aβ or tau pathology.

### 2.2. Screening of Active Ingredients and Targets of *Z. jujuba*

The TCMSP database (http://tcmspw.com) was systematically searched to identify putative bioactive compounds of *Z. jujuba*. To ensure adequate pharmacokinetic properties and potential translational relevance, candidate compounds were screened using oral bioavailability (OB) ≥ 30% and drug‐likeness (DL) ≥ 0.18 as inclusion criteria. These thresholds are widely applied in network pharmacology studies to enrich compounds with favorable intestinal absorption and structural similarity to known therapeutic agents [22]. Considering that the present study focuses on neuroinflammation‐associated cognitive impairment, the predicted blood–brain barrier (BBB) permeability values provided by the TCMSP database were additionally examined to evaluate the potential ability of candidate compounds to penetrate the central nervous system. Rather than being used as a strict filtering criterion at this stage, BBB permeability was incorporated as a supportive pharmacokinetic parameter to facilitate subsequent prioritization of compounds with potential neuropharmacological relevance in downstream analyses.

For each retained compound, the corresponding putative protein targets were retrieved from the TCMSP database. To improve data reliability and ensure consistency across downstream analyses, all target protein names were standardized to official human gene symbols using the UniProt Knowledgebase (UniProtKB; http://www.uniprot.org). During this process, the “organism” parameter was restricted to *Homo sapiens* to exclude non‐human homologs, and only reviewed entries (Swiss‐Prot) were retained to ensure high annotation accuracy.

### 2.3. Identification of Neurodegeneration‐Associated Targets Based on AD Transcriptomic Data

AD‐associated targets were identified using an integrated database‐ and transcriptome‐driven strategy. First, disease‐related genes were retrieved from the GeneCards database (https://www.genecards.org) using the keyword “AD.” To reduce background noise and enhance disease relevance, only genes with a relevance score ≥ 20 were retained for further analysis. These genes were used primarily as a broad disease annotation resource to capture molecular processes associated with neurodegeneration and cognitive dysfunction, rather than as direct representations of AD‐specific mechanisms. In parallel, transcriptomic data were obtained from the GEO database (GSE138260, platform GPL27556), which includes 19 cognitively normal control samples and 17 AD samples. Weighted gene co‐expression network analysis (WGCNA) was conducted in R (version 4.4.1) as an exploratory systems biology approach to identify gene co‐expression modules potentially associated with disease‐related transcriptional alterations. Genes were ranked according to median absolute deviation (MAD), and the bottom 50% with low expression variability were excluded to reduce noise and improve network stability. An appropriate soft‐thresholding power (*β*) was selected using the pickSoftThreshold function to approximate scale‐free topology (*R* ^2^ > 0.85). Based on the selected *β* value, an adjacency matrix was constructed and subsequently transformed into a topological overlap matrix (TOM). Hierarchical clustering was performed using the hclust function, and gene modules were identified using the dynamic tree cut algorithm (cutreeDynamic function). Modules with highly similar eigengenes were merged using the mergeCloseModules function to avoid redundancy.

Module eigengenes (MEs) were calculated to represent the principal expression pattern of each module. Module–trait relationships were then evaluated to identify modules showing consistent association trends with disease status. Considering the relatively limited sample size of the transcriptomic dataset, WGCNA was applied primarily as an exploratory co‐expression screening approach rather than as a definitive method for identifying disease‐specific genes. Therefore, modules showing relatively stronger association trends were selected as candidate modules for further analysis, as part of an exploratory screening framework rather than a statistically confirmatory selection, and subsequently intersected with GeneCards‐derived targets to enhance robustness and reduce potential bias arising from the inclusion of nonsignificant modules. To further evaluate the robustness and cognitive relevance of the identified candidate targets, an independent external dataset (GSE1297, platform GPL96) was introduced for validation. This dataset contains transcriptomic profiles derived from human hippocampal tissue, including 9 cognitively normal control samples and 22 AD samples. The hippocampus plays a critical role in learning and memory processes and is one of the brain regions most closely associated with cognitive performance and neurodegenerative changes. Compared with many large heterogeneous datasets, the relatively comparable sample size between the training dataset and the validation cohort helps maintain similar statistical characteristics and reduces potential bias arising from substantial sample imbalance. Moreover, this dataset has been widely used to investigate inflammation‐related and neuronal cell death mechanisms in neurodegenerative conditions.

Importantly, given that the primary objective of this study was to explore neuroinflammation‐associated cognitive impairment rather than classical Aβ or tau pathology, AD‐related transcriptomic datasets were used mainly as representative sources of neurodegeneration‐associated gene expression patterns and should not be interpreted as direct models of AD pathology, but rather as reference datasets capturing transcriptional features associated with neurodegeneration and cognitive dysfunction. The hippocampus‐centered validation, therefore, provides additional support that the identified targets are linked to mechanisms underlying cognitive dysfunction rather than being restricted to general AD‐associated transcriptional alterations.

### 2.4. Construction of the Compound–Target–Disease Network

The overlapping targets between *Z. jujuba*‐associated targets and disease‐related targets were defined as candidate therapeutic targets associated with cognitive impairment. To visualize and characterize the multicomponent and multitarget regulatory relationships, a compound–target–disease network was constructed using Cytoscape software (version 3.9.0). Specifically, four node files—“*Z. jujuba*,” “Compounds,” “Cross‐targets,” and “Cognitive impairment”—were generated and imported into Cytoscape. In the resulting network, nodes represented *Z. jujuba*, its bioactive compounds, the shared targets, and cognitive dysfunction–related disease context, while edges represented the interactions or associations between compounds and targets, as well as the links between targets and the disease.

### 2.5. PPI Network Diagram Construction

A PPI network was constructed to explore the interactions among the shared targets of *Z. jujuba* and cognitive impairment–related targets identified from AD‐associated datasets, used here as a proxy for neurodegeneration‐related molecular features. The STRING database (https://cn.string-db.org) was used for this analysis, with the organism restricted to *Homo sapiens*. A medium confidence interaction score (≥0.400) was applied to balance network coverage and interaction reliability, as commonly adopted in PPI‐based network analyses.

The intersecting targets between *Z. jujuba* active components and disease‐related targets were imported into STRING to generate the PPI network. The resulting interaction data were subsequently imported into Cytoscape software (version 3.9.0) for visualization and topological analysis. In the PPI network, nodes represent proteins, and edges represent functional associations between proteins, reflecting the potential biological interactions underlying the effects of *Z. jujuba* in neuroinflammation‐associated cognitive impairment.

### 2.6. GO and KEGG Enrichment Analysis

GO enrichment analysis, including biological process (BP), cellular component (CC), and molecular function (MF) categories, as well as KEGG pathway enrichment analysis, was performed to characterize the biological functions and signaling pathways associated with the shared targets. Enrichment analyses were conducted using the Database for Annotation, Visualization, and Integrated Discovery (DAVID, version 6.8).To control for multiple testing, a false discovery rate (FDR)–adjusted *p*‐value < 0.05 was applied as the threshold for statistical significance. For each GO category (BP, CC, and MF), the top 10 significantly enriched terms ranked by FDR‐adjusted *p*‐values were selected for visualization. Enrichment results were visualized using the SangerBox 3.0 online platform. In the bubble plots, fold enrichment was plotted on the *x*‐axis, the number of enriched genes (gene count) was represented by bubble size, and statistical significance was indicated by −log_10_ (FDR‐adjusted *p*‐values). For KEGG pathway analysis, significantly enriched pathways passing the same FDR threshold were identified and visualized using pathway–gene interaction networks to provide an overview of the signaling pathways potentially involved in the effects of *Z. jujuba* on neuroinflammation‐associated cognitive impairment.

### 2.7. Machine Learning and GSEA Analytics

To identify key genes potentially involved in neuroinflammation‐associated cognitive dysfunction, three complementary machine learning algorithms were implemented in R to perform feature selection. Least absolute shrinkage and selection operator (LASSO) regression was conducted using the glmnet package. Ten‐fold cross‐validation was applied to determine the optimal penalty parameter (*λ*), allowing the selection of a sparse set of informative gene features while reducing overfitting. In parallel, a random forest (RF) model was constructed using the randomForest package, consisting of 500 decision trees. Feature importance was evaluated based on the Gini impurity index to rank genes according to their contribution to classification performance. Additionally, a support vector machine (SVM) classifier with a radial basis function (RBF) kernel was developed using the e1071 package. Recursive feature elimination (RFE) was applied to iteratively remove less informative features and identify genes with the highest discriminative power.

Genes consistently identified as important across all three machine learning models were defined as robust candidate genes, thereby enhancing the reliability of feature selection through cross‐method validation. These genes were selected for downstream functional analysis. To further explore the biological significance of these core genes, GSEA was performed using the clusterProfiler package in R. The gseGO and gseKEGG functions were applied with 1000 permutations to assess the enrichment of GO terms and KEGG pathways. Enrichment plots were generated to visualize BPs and signaling pathways that were positively or negatively associated with the core genes in neurodegeneration‐associated transcriptomic profiles. It should be noted that, although AD‐related datasets were used for model training and feature selection, the machine learning and GSEA results were interpreted in the context of neuroinflammation‐associated cognitive impairment rather than as direct representations of AD‐specific pathological mechanisms.

### 2.8. Expression Profiling of Key Targets and ROC Curve Analysis

Normalized transcriptomic datasets were obtained from the GEO database. GSE138260 (19 cognitively normal control samples and 17 AD samples) was designated as the training cohort, as it provides well‐characterized neurodegeneration‐associated transcriptomic profiles suitable for initial feature screening and model construction. GSE1297, comprising nine cognitively normal control samples and 22 AD samples and derived from human hippocampal tissue, was selected as an external validation cohort to assess the reproducibility and robustness of the identified key targets in a brain region closely associated with cognitive function. Differential expression of the screened key targets was analyzed separately in the training cohort (GSE138260) and the validation cohort (GSE1297) using the “nonparametric test” module in SangerBox 3.0. Statistical significance was defined as a FDR–adjusted *p*  < 0.05. Boxplots were generated to visualize expression distributions and annotate significance levels between disease and control groups.

ROC curve analysis was subsequently performed using the pROC package (v1.18.0) in R (v4.4.1) to evaluate the discriminative performance of each key target. ROC analyses were conducted independently in the training and validation cohorts, and the area under the curve (AUC) was calculated to quantify classification performance. Genes with an AUC > 0.70 in both datasets were considered to exhibit stable discriminative potential, within the context of exploratory analysis rather than clinical diagnostic application. Given the relatively limited sample sizes of transcriptomic datasets and the sensitivity of machine learning–based feature selection to overfitting, several measures were implemented to enhance robustness. First, candidate targets were restricted to genes consistently identified across multiple machine learning algorithms rather than relying on a single model. Second, model construction and feature selection were performed exclusively in the training cohort, while all performance evaluations were independently validated in the external dataset. Importantly, the analytical framework in this study was applied primarily for robust feature prioritization and mechanistic hypothesis generation rather than for developing a clinical diagnostic model, thereby reducing the risk of overinterpretation of classification performance. It should be noted that, although AD‐related transcriptomic datasets were used in this analysis, they were primarily employed as representative sources of neurodegeneration‐associated gene expression patterns. Therefore, the identified targets and their discriminative performance were interpreted in the context of neuroinflammation‐associated cognitive impairment rather than as direct indicators of AD‐specific pathology.

### 2.9. Nomogram Construction and Exploratory Validation

To provide an integrative and intuitive visualization of the combined contribution of the identified core targets, a nomogram model was constructed using the rms package (v6.7–0) in R. The nomogram was developed based on the expression profiles of the core targets in the training cohort and was intended as an exploratory tool rather than a definitive clinical prediction model. Calibration curves were generated to assess the agreement between predicted and observed probabilities, thereby evaluating the internal consistency of the nomogram. Each core target was assigned a weighted score according to its relative contribution, and the sum of individual scores yielded an overall risk score reflecting the combined effect of multiple targets.

In addition, decision curve analysis (DCA) was performed using the rmda package (v1.6) to estimate the potential net benefit across a range of threshold probabilities. This analysis was used to comparatively assess the incremental value of integrating multiple targets rather than to infer direct clinical decision‐making utility. Given the limited sample size and the exploratory nature of the study design, all nomogram‐based analyses were interpreted in a descriptive and hypothesis‐generating context. Consistent with the overall study framework, the nomogram was designed to reflect gene expression patterns associated with cognitive dysfunction, rather than to establish a clinically applicable diagnostic model for AD.

### 2.10. Molecular Docking

Molecular docking was performed to evaluate the binding affinity and interaction patterns between key active constituents of *Z. jujuba* and the identified core target proteins. Docking simulations were conducted using AutoDock Vina (version 1.2.3). Ligand structures were obtained from public chemical databases and subsequently geometry‐optimized in ChemBio3D using the MM2 force field to achieve energetically favorable conformations. To enhance the biological relevance of the docking analysis in the context of neuroinflammation‐associated cognitive impairment, a secondary filtering step based on BBB permeability was applied at this stage. Specifically, only compounds with a predicted logBB value > 0.3 were retained for molecular docking, indicating relatively strong BBB penetration capability and potential central nervous system bioavailability. This step was implemented to prioritize compounds more likely to exert direct effects within the brain, while acknowledging that the in vivo pharmacological effects of the whole extract cannot be attributed to these compounds alone.

Three‐dimensional structures of target proteins were retrieved from the Protein Data Bank (PDB), restricted to *Homo sapiens* entries with a resolution of ≤2.5 Å to ensure structural reliability. Protein information was further cross‐validated using reviewed entries in the UniProt database. Before docking, protein structures were prepared using PyMOL (version 2.6.0) by removing crystallographic water molecules and co‐crystallized ligands, followed by the addition of polar hydrogens. Both ligand and protein structures were converted to PDBQT format using AutoDockTools (version 1.5.7). Semi‐flexible docking was performed, with ligands treated as flexible and receptors as rigid. The docking grid box was defined to fully encompass the active binding region of each target protein. The exhaustiveness parameter was set to 15 [23], while all other parameters were maintained at default settings. For each ligand–target pair, the conformation with the lowest binding free energy (Δ*G*) was selected as the optimal binding pose. A threshold of Δ*G* ≤ −5.0 kcal/mol was applied to indicate favorable binding interactions. The docking results and intermolecular interactions, including hydrogen bonds and hydrophobic contacts, were visualized using PyMOL (version 2.6.0).

### 2.11. Chromosome and Neuroanatomical Localization

Human gene sequences, structural information, and functional annotations were obtained from the GENCODE database (version 42). Gene identifiers were standardized and annotated using R packages, including clusterProfiler, org. Hs. eg, db, and RCircos. The core gene set was mapped onto human chromosomes to visualize their genomic distribution patterns. To further investigate the neuroanatomical relevance of these core genes, spatial expression profiles were analyzed using the Human Protein Atlas (HPA, version 24.0). Protein and transcript expression data were extracted from key brain regions associated with cognitive function, including the prefrontal cortex, hippocampus, and amygdala. Comparative analysis of expression patterns across these regions was conducted to characterize the spatial distribution of core targets within the central nervous system. This analysis was designed to provide an anatomical context for the potential involvement of core genes in neuroinflammation‐associated cognitive dysfunction. Although AD‐related datasets were used for target identification, the neuroanatomical interpretation focused on general cognitive function–related brain regions rather than on AD‐specific pathological localization, thereby providing supportive anatomical context without implying disease‐specific spatial pathology.

### 2.12. Experimental Animals, Grouping, and Model Establishment

Fifty healthy male C57BL/6 mice (6–8 weeks old, 25 ± 2 g) were purchased from the Animal Experiment Center of Southwest Medical University (Luzhou, China). Animals were housed under standard laboratory conditions (22 ± 2°C, 55% ± 5% humidity, 12 h light/dark cycle) with free access to food and water. After a 7‐day acclimatization period, mice were randomly assigned into five groups (*n* = 10 per group): control group: oral gavage with 0.9% saline for 7 days + intraperitoneal (i.p.) injection of saline on days 4–8; scopolamine model group: oral gavage with 0.9% saline for 7 days + i.p. injection of scopolamine hydrobromide (1 mg/kg) on days 4–8; low‐dose *Z. jujuba* suspension group (L‐Js): oral administration of *Z. jujuba* suspension (100 mg/kg/day) for 7 days + i.p. injection of scopolamine (1 mg/kg) on days 4–8; high‐dose *Z. jujuba* suspension group (H‐Js): oral administration of *Z. jujuba* suspension (200 mg/kg/day) for 7 days + i.p. injection of scopolamine (1 mg/kg) on days 4–8; positive control group (donepezil [DOP]): oral administration of DOP hydrochloride (5 mg/kg/day) for 7 days + i.p. injection of scopolamine (1 mg/kg) on days 4–8. Group allocation and outcome assessments were performed in a randomized manner, and behavioral analyses were conducted by investigators blinded to group assignments.

Scopolamine hydrobromide (batch no. H51022122; Nordina, China) was dissolved in sterile saline and administered intraperitoneally 1 h after oral gavage for five consecutive days to induce transient cognitive impairment via cholinergic dysfunction, which is a widely used pharmacological model for learning and memory deficits [24]. Importantly, scopolamine was administered only to the model and treatment groups, whereas control mice received an equivalent volume of saline. The *Z. jujuba* suspension used in this study was prepared by the Chinese Medicine Pharmacy of the Affiliated Hospital of Southwest Medical University. Briefly, authenticated dried fruits of *Z. jujuba* Mill. (product name and voucher number provided in Supporting Information 1: Table S1) were subjected to two rounds of hot‐water extraction (solid–liquid ratio 1:15, w/v). The combined extracts were filtered, concentrated, and homogenized to yield a stable brownish‐red translucent suspension containing water‐soluble phytochemicals and colloidal fractions. All batches were prepared using the same raw material source and standardized decoction procedures to ensure batch‐to‐batch consistency and quality stability across experiments, and the suspension exhibited stable physical characteristics without visible precipitation or phase separation during storage.

The detailed preparation procedure, representative chemical composition, and bioavailability considerations are provided in Supporting Information 2. Although chromatographic characterization (e.g., HPLC or LC–MS/MS profiling) was not performed in the present study, the preparation method follows standardized aqueous extraction procedures widely reported for *Z. jujuba*, and the resulting chemical composition is consistent with well‐established phytochemical profiles documented in previous studies. Therefore, the compositional reliability of the extract is supported by methodological standardization and extensive prior evidence. Furthermore, as a typical food–medicine homology substance, *Z. jujuba* is commonly administered as a whole‐extract preparation in both experimental pharmacology and clinical practice, where biological activity is attributed to the synergistic effects of multiple components rather than individual compounds. Accordingly, the present study focuses on the pharmacological evaluation of the whole extract, and the level of chemical characterization employed is considered sufficient to support interpretation of the observed biological effects. In this context, the in vivo pharmacological outcomes are interpreted as the integrated effects of a multicomponent system, rather than direct validation of individual compound–target interactions predicted by molecular docking.

DOP hydrochloride tablets (batch no. H20070181; Shandong Buchang Pharmaceutical Co., Ltd. China) were finely ground and dissolved in saline before use. The selected dose of DOP (5 mg/kg) was based on previous studies demonstrating its efficacy in ameliorating scopolamine‐induced cognitive deficits in mice [25] and was used as a pharmacological positive control. Dose selection for *Z. jujuba* was determined according to the human–mouse body surface area conversion method and previous pharmacological studies [26, 27]. Although the precise in vivo concentrations of individual constituents were not quantified, previous studies have demonstrated that major components of *Z. jujuba*, including flavonoids, polysaccharides, and triterpenoids, exhibit measurable OB and central nervous system activity, supporting the biological plausibility of the dosing regimen used.

Only male mice were included to minimize variability related to sex‐dependent hormonal fluctuations and to ensure experimental consistency. Potential sex‐associated differences warrant further investigation in future studies. All animal procedures were approved by the Animal Experiment Ethics Committee of Southwest Medical University (Approval No. 20221026‐013) and were conducted in accordance with national regulations and international guidelines, including the ARRIVE 2.0 guidelines [28]. Animals were handled under appropriate anesthesia, and euthanasia was performed by i.p. injection of an overdose of pentobarbital sodium (150 mg/kg) to minimize suffering. It should be noted that the scopolamine‐induced model reflects transient cholinergic dysfunction–associated cognitive impairment rather than the full spectrum of AD pathology and does not recapitulate core pathological features such as Aβ deposition or tau hyperphosphorylation. Therefore, the in vivo findings are interpreted in the context of cognitive function modulation rather than as direct evidence of AD‐specific mechanisms. It is important to note that the in vivo validation focuses on assessing the pharmacological modifiability of PTGS2‐centered pathways identified from AD transcriptomes, rather than direct validation of compound–target interactions predicted by molecular docking. The crude extract administered in vivo contains a complex matrix of constituents, and the observed effects likely reflect emergent properties of the whole system rather than specific ligand–protein binding events modeled in silico.

### 2.13. Behavioral Assessment

The MWM test was performed to evaluate spatial learning and memory in mice, as previously described [29]. The apparatus consisted of a circular pool (diameter: 120 cm; height: 50 cm) filled with water maintained at 22 ± 1°C. The pool was divided into four equal quadrants, and a circular escape platform (diameter: 10 cm) was submerged 1 cm below the water surface and fixed at the center of quadrant III. To render the platform invisible, nontoxic milk powder was added to the water. Animal movement was recorded and analyzed using an automated video tracking system (Med Associates, USA). Mice were allowed to acclimate to the testing environment for 1 day before the experiment. The acquisition (training) phase was conducted over five consecutive days, with three trials per day. In each trial, mice were gently placed into the pool from pseudo‐randomly assigned starting points (quadrants I, II, or IV), facing the pool wall. Each mouse was allowed a maximum of 60 s to locate the hidden platform. If a mouse failed to find the platform within the allotted time, it was gently guided to the platform and allowed to remain there for 10 s. The escape latency (time to reach the platform) was recorded as an index of spatial learning ability, and the average value across trials was used for statistical analysis.

On Day 6, a probe trial was conducted to assess memory retention. The platform was removed, and mice were released from quadrant I. Each mouse was allowed to swim freely for 120 s, during which the number of platform crossings (former platform location) and swimming trajectories were recorded. In addition, the time spent in the target quadrant was quantified as a complementary indicator of spatial memory performance. These parameters were used to evaluate spatial memory performance. All behavioral tests were conducted during the same time period each day to minimize circadian variability, and the experimenters were blinded to group allocation during data acquisition and analysis.

### 2.14. Histopathological Examination (HE Staining)

Following behavioral testing, mice were euthanized as described earlier, and brain tissues were rapidly collected. The hippocampal regions were dissected and fixed in 4% paraformaldehyde for 24–48 h at room temperature. After fixation, tissues were dehydrated through a graded ethanol series (70%, 80%, 90%, 95%, and 100%; 1–2 h per step), cleared in xylene, and embedded in paraffin. Paraffin‐embedded tissues were sectioned into 4–5 μm thick slices using a rotary microtome. Sections were deparaffinized in xylene, rehydrated through descending concentrations of ethanol, and rinsed in distilled water. Hematoxylin staining was performed for 5–10 min to visualize cell nuclei, followed by eosin staining for 2–5 min to stain cytoplasmic components. After differentiation and thorough washing, sections were dehydrated, cleared in xylene, and mounted with neutral resin and coverslips.

Histopathological changes in the hippocampus were examined under a light microscope (×200 and ×400 magnification). Representative images were captured from the CA1 region of the hippocampus for comparative analysis. Neuronal arrangement, cellular morphology, and structural integrity were evaluated to assess cognitive impairment–associated neuronal damage induced by scopolamine and the potential neuroprotective effects of *Z. jujuba* treatment. For semi‐quantitative assessment, neuronal damage was evaluated based on cell density, nuclear condensation, and structural disorganization in a blinded manner. At least three nonoverlapping fields per section and three sections per animal were analyzed to ensure reproducibility.

### 2.15. Western Blot Analysis

Following completion of behavioral assessments, mice were euthanized in accordance with approved ethical protocols. Hippocampal tissues were rapidly dissected on ice, rinsed with pre‐cooled phosphate‐buffered saline (PBS), and homogenized in radioimmunoprecipitation assay (RIPA) lysis buffer supplemented with protease and phosphatase inhibitors (Beyotime, China). Tissue homogenization was performed on ice using a mechanical tissue grinder, followed by brief ultrasonic disruption (10 cycles). Lysates were incubated on ice for 45 min to ensure complete protein extraction and subsequently centrifuged at 12,000 × *g* for 15 min at 4°C. The supernatants were collected as total protein extracts. Protein concentrations were determined using a bicinchoninic acid (BCA) protein assay kit (Beyotime, China). Equal amounts of protein (20 μg per lane) were mixed with 5× SDS loading buffer, denatured at 100°C for 10 min, and separated by SDS–polyacrylamide gel electrophoresis (SDS–PAGE) using 10% resolving gels and 4% stacking gels. Electrophoresis was conducted at 80 V for 15 min, followed by 120 V for 60 min. Proteins were then transferred onto polyvinylidene difluoride (PVDF) membranes using a wet‐transfer system at 250 mA for 80 min. Membranes were blocked with 5% non‐fat milk in tris‐buffered saline containing 0.1% Tween‐20 (TBST) for 1.5 h at room temperature, followed by overnight incubation at 4°C with the following primary antibodies: PTGS2 (1:1500, rabbit polyclonal, Beyotime, AF1924); NOD‐like receptor (NLR)P3 (1:1500, rabbit polyclonal, Beyotime, AF2155); IL‐1β (1:1500, rabbit polyclonal, Beyotime, AF7209); NF‐κB p65 (1:1500, rabbit polyclonal, Beyotime, AF0246); p38 MAPK (1:1500, rabbit polyclonal, Beyotime, AF7668). These targets were selected based on their established roles in neuroinflammation‐related signaling pathways, including the NF‐κB and MAPK pathways.

After washing three times with TBST (10 min each), membranes were incubated with HRP‐conjugated goat anti‐rabbit IgG secondary antibody (1:5000, Beyotime, A0208) for 1 h at room temperature. Protein bands were visualized using an enhanced chemiluminescence (ECL) detection kit and captured with a ChemiDoc XRS + imaging system (Bio‐Rad, USA). Band intensities were quantified using ImageJ software (National Institutes of Health, USA) and normalized to β‐actin as the internal loading control. The stability of β‐actin expression across groups was verified before normalization. To ensure accuracy, all samples were processed under identical experimental conditions, and exposure times were kept within the linear detection range. Western blot analyses were performed with at least three independent biological replicates per group, and each biological replicate was analyzed in technical duplicate to ensure reproducibility. Quantitative results are expressed as mean ± standard deviation (SD). All quantitative analyses were performed in a blinded manner to minimize bias. In addition, representative full‐length blots are provided in the Supporting Information (Supporting Information 3: Figure S1) to ensure transparency and data integrity. Statistical analysis of western blot data was conducted as described in Section 2.16.

### 2.16. Statistical Analysis

All statistical analyses were performed using GraphPad Prism version 9.0 (GraphPad Software, USA). Data are presented as mean ± SD unless otherwise specified. Behavioral data obtained from the MWM acquisition phase were analyzed using repeated‐measures one‐way analysis of variance (ANOVA) to assess learning performance across training days. Data from other experiments were analyzed using one‐way ANOVA followed by Tukey’s post hoc test for multiple comparisons. Normality of data distribution was assessed before statistical analysis. A two‐tailed *p*‐value < 0.05 was considered statistically significant.

## 3. Results

### 3.1. Main Active Ingredients and Targets of *Z. jujuba*

Based on the TCMSP database, a total of 29 bioactive compounds of *Z. jujuba* were identified according to the predefined screening criteria (OB ≥ 30% and DL ≥ 0.18). These compounds included stepharine, spiradine A, jujuboside A, coumestrol, and daechuine S6, among others (Supporting Information 1: Table S1). In addition, BBB permeability‐related parameters provided by the TCMSP database were examined to preliminarily assess the potential central nervous system relevance of the identified compounds and to inform subsequent prioritization in downstream analyses. Correspondingly, 183 putative protein targets associated with these compounds were obtained from the TCMSP database and subsequently standardized using the UniProt database, followed by the removal of duplicate entries.

### 3.2. Identification of Neurodegeneration‐Associated Targets Based on Transcriptomic Analysis

**Figure 2:**
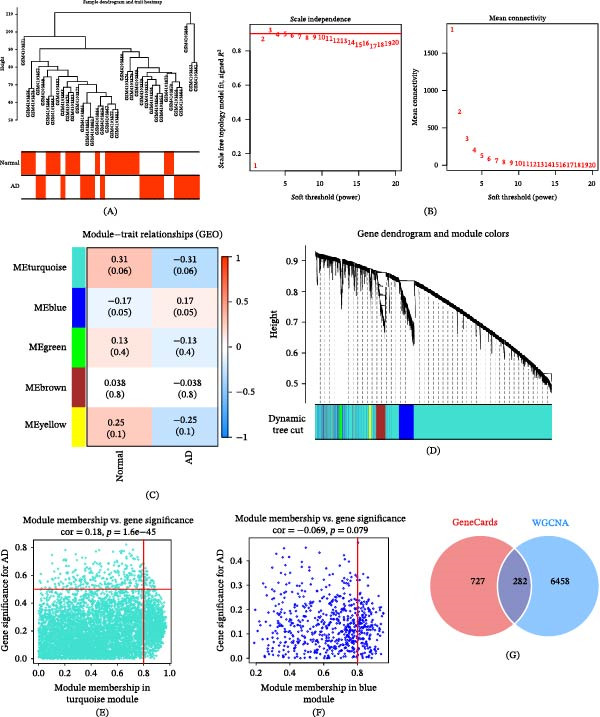
Identification of cognitive impairment–related gene modules based on AD‐derived transcriptomic data using weighted gene co‐expression network analysis (WGCNA). (A) Sample clustering dendrogram and trait heatmap. (B) Scale‐free topology analysis. (C) Gene clustering dendrogram and module assignment. (D) Module–trait relationship heatmap based on AD status. (E, F) Relationships between module membership and gene significance in key modules. (G) Venn diagram showing the intersection of WGCNA‐derived module genes with AD‐associated targets from GeneCards, yielding candidate targets related to cognitive dysfunction. AD datasets were used as a proxy to capture cognitive impairment–related transcriptional features, and module selection was applied as an exploratory screening step.

A total of 1009 AD–associated targets were first retrieved from the GeneCards database based on the predefined screening criteria described in the Methods section. These targets were used as a reference resource to capture neurodegeneration‐associated molecular features, rather than being interpreted as direct indicators of AD‐specific pathology. To further refine disease‐relevant targets at the transcriptomic level, WGCNA was performed using the GEO dataset GSE138260 in R (version 4.4.1). After sample quality control, a sample clustering heatmap was generated to assess dataset stability (Figure 2A). The soft‐thresholding power was determined using the scale‐free topology criterion, and *β* = 3 was selected as the optimal value to construct a scale‐free co‐expression network (scale‐free topology fit index *R* ^2^ = 0.90; Figure 2B). Gene modules were identified by hierarchical clustering and dynamic tree cutting, followed by module merging based on eigengene similarity (Figure 2C,D).

Module–trait relationship analysis was performed to identify gene modules associated with disease‐related transcriptional alterations. Among all identified co‐expression modules, the turquoise module (MEturquoise) displayed the strongest negative correlation with disease status (*r* = −0.31, *p* = 0.06), whereas the blue module (MEblue) exhibited the highest positive correlation (*r* = 0.17, *p* = 0.05) (Figure 2E,F). Although these correlations did not reach the conventional statistical significance threshold (*p*  < 0.05), it should be noted that WGCNA in this study was applied as an exploratory systems biology approach to identify co‐expression patterns rather than to establish statistically definitive associations. Therefore, the selected modules should be interpreted as hypothesis‐generating rather than confirmatory. Given the relatively limited sample size of the dataset, modules were prioritized based on correlation strength and biological plausibility, which has been widely adopted in exploratory transcriptomic analyses to capture functionally relevant gene networks. Accordingly, the MEturquoise and MEblue modules were selected for downstream analyses, yielding a total of 6740 genes (6091 genes from MEturquoise and 649 genes from MEblue). To further enhance robustness and reduce potential false positives, genes derived from these WGCNA modules were intersected with AD‐associated targets retrieved from the GeneCards database (relevance score ≥ 20) using Venny 2.1.0. This integrative strategy resulted in 282 candidate targets (Figure 2G), representing a refined gene set supported by both co‐expression patterns and curated disease relevance.

Importantly, considering that AD‐related transcriptomic datasets were used in this study primarily as representative sources of neurodegeneration‐associated gene expression patterns, the identified targets were interpreted in the context of neuroinflammation‐associated cognitive dysfunction rather than as direct indicators of AD‐specific pathology.

### 3.3. Construction of the Compound–Target Network Based on Disease‐Associated Targets

**Figure 3:**
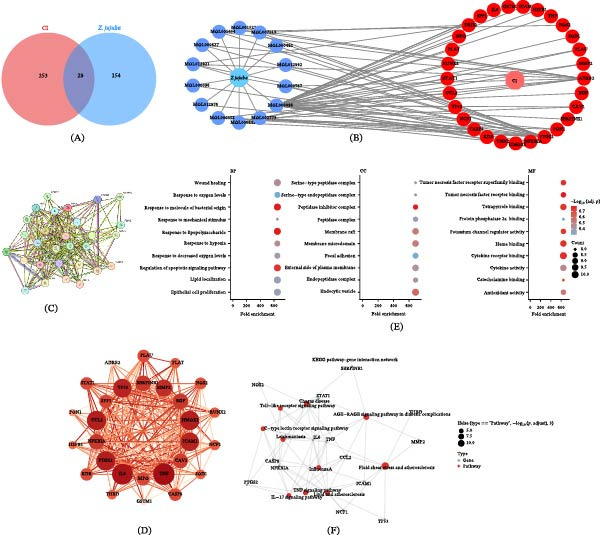
Identification of shared targets between *Z. jujuba* and cognitive impairment–related targets and functional enrichment analysis. (A) Venn diagram showing overlapping targets between *Z. jujuba*‐related targets and AD‐derived cognitive dysfunction–associated targets. (B) Compound–target network illustrating interactions between *Z. jujuba* bioactive components and shared targets. (C, D) Protein–protein interaction (PPI) network highlighting hub genes. (E) GO enrichment analysis (BP, CC, and MF). (F) KEGG pathway enrichment and gene–pathway interaction network, suggesting involvement in inflammation‐related signaling processes associated with cognitive impairment.

An intersection analysis was performed using the MicroBioinformatics online platform to identify shared targets between the predicted targets of *Z. jujuba* active constituents and disease‐associated targets derived from neurodegeneration‐related datasets. These overlapping targets were defined as putative regulatory targets potentially involved in neuroinflammation‐associated cognitive dysfunction (Figure 3A). Subsequently, Cytoscape software was employed to construct a compound–target regulatory network, illustrating the interactions between *Z. jujuba* active components and the shared targets. The resulting network comprised 14 bioactive components and 29 shared targets (Figure 3B), providing a systems‐level overview of the multicomponent and multitarget characteristics of *Z. jujuba*.

### 3.4. PPI Network Construction and Hub Gene Identification

A PPI network was constructed using the STRING database based on the shared targets between *Z. jujuba* and AD‐associated gene sets, yielding a network comprising 29 nodes and 231 edges (Figure 3C). The interaction network was subsequently imported into Cytoscape for visualization and topological analysis. Network centrality parameters, including degree centrality, were calculated using the NetworkAnalyzer plugin. Degree centrality was selected as the primary topological metric to evaluate node importance within the network, as it reflects the number of direct interactions and has been widely applied in PPI‐based network pharmacology studies. The top 10 genes ranked by degree centrality—TNF, IL‐6, PTGS2, HMOX1, CCL2, ICAM1, TP53, MMP2, NFKBIA, and SERPINE1—were identified as hub genes, suggesting their potential importance in the regulation of the interaction network, while not implying direct causal roles in disease processes. A core target interaction subnetwork was further visualized (Figure 3D), in which node size and color intensity were proportional to degree values, reflecting the relative topological importance of each hub gene. It should be noted that hub gene identification in this study is based on network topology and is intended for prioritization of candidate targets rather than definitive evidence of biological causality.

### 3.5. GO and KEGG Enrichment Analysis

GO enrichment analysis was performed to characterize the functional features of the shared targets. In the BP category, the targets were predominantly enriched in hypoxia‐related processes, including response to hypoxia and response to decreased oxygen levels. For the CC category, enriched terms were mainly associated with functional structures such as the peptidase inhibitor complex and endocytic vesicles. In the MF category, the shared targets were significantly enriched in cytokine receptor binding and TNF receptor binding, suggesting potential involvement in inflammation‐ and immune‐related signaling activities. The GO enrichment results were visualized using bubble plots generated by SangerBox 3.0, in which fold enrichment, gene count, and statistical significance (−log_10_ FDR‐adjusted *p*‐values) were simultaneously displayed (Figure 3E).

KEGG pathway enrichment analysis was subsequently performed using the same gene set and statistical threshold (FDR‐adjusted *p*  < 0.05). The significantly enriched pathways were mainly related to inflammation‐ and immune‐associated signaling pathways, including the TNF signaling pathway, AGE–RAGE signaling pathway in diabetic complications, and Toll‐like receptor signaling pathway. KEGG enrichment results were visualized using SangerBox 3.0, and pathway–gene interaction networks were constructed to provide an overview of the potential signaling pathways associated with the identified targets (Figure 3F), rather than to imply direct causal relationships.

### 3.6. Machine Learning–based Identification of Core Targets and GSEA Analysis

**Figure 4:**
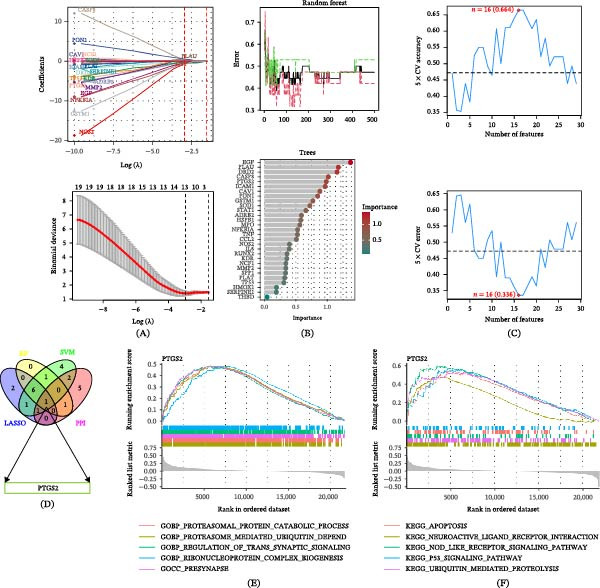
Machine learning–based identification of PTGS2 as a core target and enrichment analysis. (A) Least absolute shrinkage and selection operator (LASSO) regression analysis for feature selection. (B) Random forest analysis identifying high‐importance candidate targets. (C) Support vector machine–recursive feature elimination (SVM–RFE) analysis for target prioritization. (D) Venn diagram showing the intersection of core targets identified by LASSO, random forest, SVM–RFE, and protein–protein interaction network analysis, highlighting PTGS2 as the shared key target. (E) GO‐based GSEA of PTGS2‐associated gene signatures in AD transcriptomes. (F) KEGG pathway‐based GSEA of PTGS2‐associated gene signatures in AD transcriptomes, indicating enrichment of inflammation‐ and neurodegeneration‐related pathways.

LASSO regression, optimized by 10‐fold cross‐validation, identified 11 candidate genes with non‐zero coefficients, including PTGS2, DRD2, and NOS2 (Figure 4A; Supporting Information 1: Table S1). The RF model ranked gene importance based on the mean decrease in Gini index, and the top nine genes with importance scores ≥ 0.75 were selected (Figure 4B; Supporting Information 1: Table S1). In parallel, SVM–RFE identified 16 genes, including PTGS2, PLAT, and NFKBIA, by iteratively eliminating features with minimal contribution to classification performance (Figure 4C; Supporting Information 1: Table S1). To identify robust core targets, the results of multiple screening strategies were integrated, including three machine learning algorithms (LASSO regression, RF, and SVM–RFE) and topological analysis of the PPI network. Specifically, the top‐ranked genes from each machine learning model, together with the top 10 genes with the highest degree centrality in the PPI network, were intersected. This conservative integration strategy was applied to enhance the robustness of feature selection and to reduce potential model‐specific bias.

As a result, only one gene, PTGS2, was consistently identified across all four approaches (Figure 4D). The identification of a single overlapping gene reflects the stringency of the selection criteria rather than a lack of biological relevance and highlights PTGS2 as a highly stable candidate across complementary analytical frameworks. GSEA was subsequently performed to explore BPs and pathways associated with PTGS2. GSEA–GO analysis revealed significant enrichment in processes related to proteasomal protein catabolism and synaptic organization, including GOBP_PROTEASOMAL_PROTEIN_CATABOLIC_PROCESS and GOCC_PRESYNAPSE (Figure 4E). GSEA–KEGG analysis further indicated enrichment of inflammation‐ and stress‐related signaling pathways, such as the NLR signaling pathway and the p53 signaling pathway (Figure 4F).

### 3.7. ROC Curve and Diagnostic Nomogram of the Core Target

**Figure 5:**
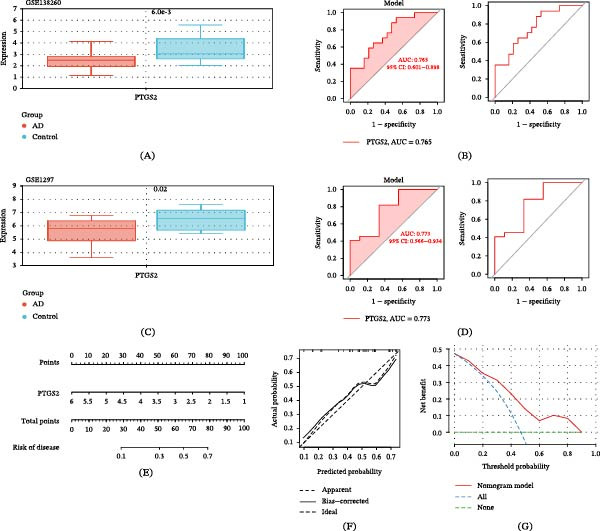
Expression and diagnostic performance of the core target PTGS2. (A, C) Expression levels of PTGS2 in the training dataset (GSE138260) and external validation dataset (GSE1297). (B, D) Receiver operating characteristic (ROC) curve analysis evaluating the diagnostic performance of PTGS2 in the two datasets. (E) Nomogram model constructed based on PTGS2 for predicting cognitive impairment–related status. (F) Calibration curve assessing the agreement between predicted and observed outcomes. (G) Decision curve analysis (DCA) evaluating the potential clinical utility of the PTGS2‐based model.

The GSE138260 dataset was used as the training cohort to evaluate the expression level of the identified core target, PTGS2, between control and AD groups (Figure 5A). The results showed that PTGS2 expression was significantly upregulated in the AD group compared with controls. ROC curve analysis demonstrated that PTGS2 exhibited favorable discriminative performance in the training dataset, with an AUC of 0.765 (model, 95% CI: 0.601–0.898) (Figure 5B), indicating a moderate discriminative capacity for distinguishing transcriptomic patterns associated with cognitive dysfunction. To further assess the robustness of this finding, the independent dataset GSE1297 was used as an external validation cohort. Consistent with the training dataset, PTGS2 expression remained significantly elevated in the AD group (Figure 5C). ROC curve analysis in the validation dataset yielded an AUC of 0.773 (model, 95% CI: 0.566–0.934) (Figure 5D), suggesting stable discriminative performance across independent cohorts. Based on the expression profile of PTGS2, a diagnostic nomogram was constructed to provide an intuitive visualization of its potential discriminative contribution (Figure 5E). The calibration curve demonstrated good agreement between predicted and observed probabilities, indicating acceptable model consistency (Figure 5F). In addition, DCA showed that the PTGS2‐based model provided a higher net benefit across a range of threshold probabilities compared with the “treat‐all” or “treat‐none” strategies (Figure 5G). It should be noted that, given the limited sample size and the single‐gene nature of the model, the nomogram is intended as an exploratory tool for evaluating discriminative potential rather than a clinically applicable diagnostic model. Furthermore, these results reflect transcriptomic differences associated with neuroinflammation‐related cognitive dysfunction and should not be interpreted as direct diagnostic or predictive evidence for AD.

### 3.8. Molecular Docking

**Figure 6:**
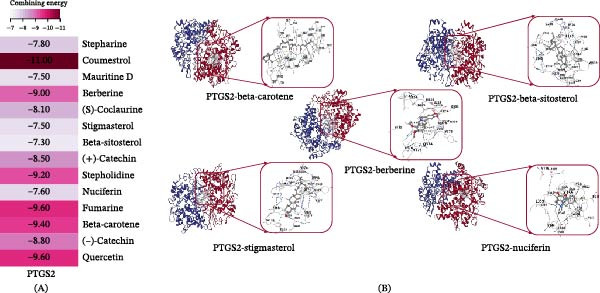
Molecular docking analysis of *Z. jujuba* bioactive compounds with PTGS2. (A) Heatmap of binding free energies (Δ*G*) for 14 key bioactive compounds from *Z. jujuba* docked with PTGS2, indicating generally strong and stable interactions. (B) Representative three‐dimensional docking conformations of high‐affinity compound–PTGS2 complexes. PTGS2 is shown as a ribbon model, while compounds are displayed as stick models. Hydrogen bond interactions between ligands and key residues within the PTGS2 binding pocket are indicated. Compounds with predicted blood–brain barrier permeability (logBB > 0.3) were prioritized for visualization, though the in vivo effects likely reflect integrated actions of multiple constituents.

Molecular docking was performed to evaluate the binding interactions between representative active constituents of *Z. jujuba* and the core target PTGS2. Based on the compound–target network analysis, 14 active constituents directly associated with PTGS2 were initially identified and selected for docking analysis (Figure 6A). Binding affinity was assessed according to the predicted binding free energy (Δ*G*), with lower (more negative) values indicating stronger and more stable ligand–target interactions. All 14 compounds exhibited favorable binding energies toward PTGS2, with an average Δ*G* of approximately −8.5 kcal/mol, suggesting potential interaction capacity under in silico conditions rather than confirming direct biological interactions. To further improve the biological relevance of the docking results in the context of neuroinflammation‐associated cognitive impairment, an additional filtering step based on predicted BBB permeability was applied. Compounds with logBB > 0.3 were considered to possess higher potential for central nervous system exposure. Among the 14 PTGS2‐associated compounds, five constituents—beta‐carotene, stigmasterol, beta‐sitosterol, nuciferine, and berberine—met this criterion and were, therefore, selected for detailed structural visualization and interaction analysis (Figure 6B). However, it should be emphasized that the in vivo effects of *Z. jujuba* likely reflect the integrated activity of multiple constituents, including those with lower predicted BBB penetration, rather than being solely attributable to the five visualized compounds.

### 3.9. Chromosome and Neuroanatomical Localization

**Figure 7:**
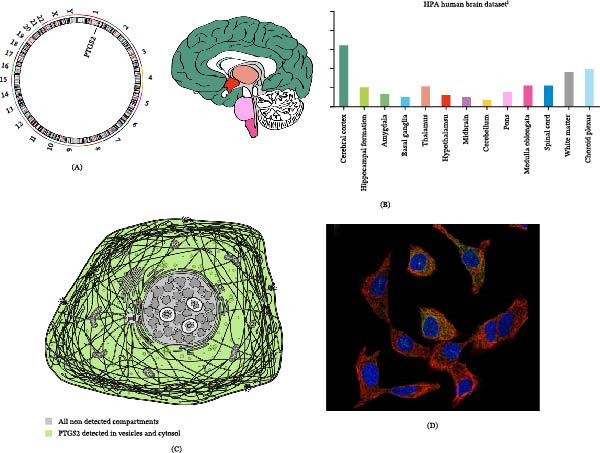
Spatial localization characteristics of PTGS2 relevant to cognitive impairment. (A) Chromosomal localization. (B) Neuroanatomical expression distribution across brain regions. (C, D) Cellular and subcellular localization patterns based on database annotation, indicating vesicular localization associated with inflammatory signaling processes.

Chromosomal localization analysis revealed that PTGS2 is mapped to chromosome 1, as illustrated in Figure 7A. To further investigate its neuroanatomical relevance, tissue‐ and brain region–specific expression patterns of PTGS2 were analyzed using data from the HPA database. The results indicated that PTGS2 is expressed across multiple brain regions, with relatively higher expression levels observed in the cerebral cortex (Figure 7B), suggesting its potential involvement in higher‐order cognitive functions, although functional validation in specific contexts is still required. At the cellular level, subcellular localization data from the HPA database demonstrated that PTGS2 is predominantly localized in vesicular structures, consistent with its reported role in inflammatory mediator synthesis and intracellular signaling processes (Figure 7C,D). These findings provide anatomical and subcellular context supporting the involvement of PTGS2 in neuroinflammation‐associated cognitive dysfunction.

### 3.10. Results of Behavioral Experiments

**Figure 8:**
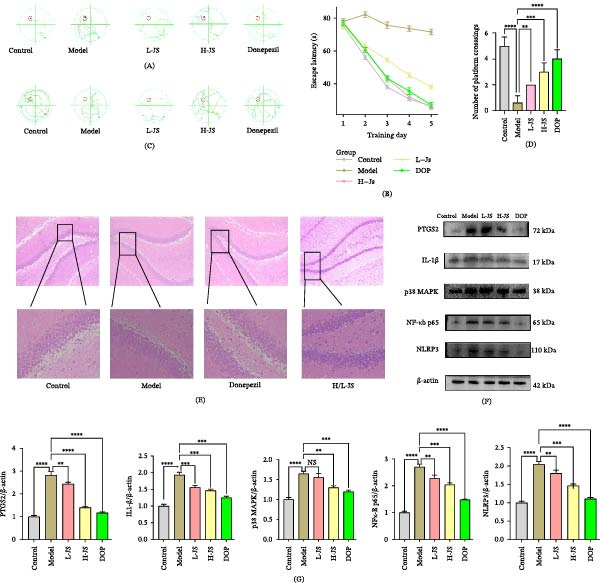
Effects of *Z. jujuba* on cognitive impairment, hippocampal morphology, and neuroinflammatory signaling in a scopolamine‐induced cognitive impairment mouse model. (A) Representative swimming trajectories during the acquisition phase of the Morris water maze (MWM). (B) Learning curve (line graph) showing escape latency to reach the hidden platform from Day 1 to Day 5. (C) Representative swimming trajectories during the probe trial. (D) Number of platform crossings during the probe trial, reflecting spatial memory retention. (E) Representative hematoxylin–eosin (HE) staining of the hippocampal CA1 region in control, model, donepezil (DOP), low‐dose *Z. jujuba* suspension (L‐JS), and high‐dose *Z. jujuba* suspension (H‐JS) groups. (F) Representative western blot bands of neuroinflammatory markers, including PTGS2, IL‐1β, p38 MAPK, NLRP3, and NF‐κB p65, with β‐actin as an internal control. (G) Densitometric quantification of western blot results, normalized to β‐actin, presented as mean ± SD. All behavioral, histological, and molecular analyses were conducted under blinded conditions. β‐actin expression stability was verified before normalization.  ^∗∗^ *p* < 0.01;  ^∗∗∗^ *p* < 0.001;  ^∗∗∗∗^ *p* < 0.0001.

During the acquisition phase of the MWM test (representative swimming trajectories shown in Figure 8A and escape latency summarized in Figure 8B), escape latency progressively decreased across training days in all groups, indicating effective learning over time. Mice in the model group exhibited significantly prolonged escape latencies compared with the control group from Day 2 onward (*p*  < 0.01), reflecting impaired spatial learning ability induced by scopolamine. No significant differences were observed among groups on Day 1 (*p*  > 0.05), confirming comparable baseline performance prior to training. From Day 2 to Day 5, mice in the control, DOP‐treated, low‐dose *Z. jujuba* suspension (L‐Js), and high‐dose *Z. jujuba* suspension (H‐Js) groups all showed significantly reduced escape latencies compared with the model group on corresponding days (*p*  < 0.01). Among these, the DOP group exhibited the most pronounced improvement, followed by the H‐Js group, indicating a relatively stronger effect on spatial learning performance under the current experimental conditions.

In the spatial probe trial (representative swimming trajectories shown in Figure 8C; number of platform crossings shown in Figure 8D), mice in the model group crossed the former platform location significantly fewer times than those in the control group (*p*  < 0.01), indicating impaired spatial memory retention. Treatment with DOP and *Z. jujuba* suspension significantly increased the number of platform crossings compared with the model group (*p*  < 0.01). Notably, the DOP group exhibited the highest number of platform crossings, while the H‐Js group also showed a marked but comparatively moderate improvement, consistent with its effect observed during the acquisition phase.

### 3.11. Pathological (HE Staining) Experiments

As shown in Figure 8E, HE staining of hippocampal sections revealed that neurons in the CA1 region of the control group exhibited normal morphological characteristics, including clear cellular outlines, abundant cytoplasm, and well‐defined nuclei. Neurons were densely distributed and arranged in an orderly manner, with no evident histopathological abnormalities. In contrast, the model group displayed apparent neuronal damage in the CA1 region, characterized by disorganized cellular arrangement, reduced neuronal density, and morphological alterations such as nuclear pyknosis and cytoplasmic shrinkage, indicating neuronal injury following scopolamine administration. Compared with the model group, both the DOP‐treated group and the *Z. jujuba*–treated groups (L‐Js and H‐Js) exhibited attenuated histopathological alterations, as reflected by relatively preserved neuronal morphology and improved cellular organization. In the *Z. jujuba*–treated groups, neurons in the CA1 region appeared more regularly arranged and better preserved than those in the model group, although mild structural abnormalities were still observed in some cells. No obvious difference in histopathological improvement was observed between the low‐ and high‐dose *Z. jujuba* groups under the present experimental conditions. These observations suggest a protective effect of *Z. jujuba* on hippocampal neuronal structure in this model.

### 3.12. Protein Immunoblotting Western Blotting

As shown in Figure 8F,G, western blot analysis demonstrated that the protein expression levels of pro‐inflammatory mediators, including PTGS2, IL‐1β, NF‐κB p65, and NLRP3, were significantly increased in the model group compared with the control group (*p*  < 0.05). In contrast, treatment with DOP and *Z. jujuba* (L‐Js and H‐Js) resulted in significant downregulation of these proteins, supporting a potential modulatory effect on neuroinflammation‐related signaling pathways relative to the model group (*p*  < 0.05). Among the *Z. jujuba*–treated groups, the H‐Js group exhibited a more pronounced inhibitory effect on PTGS2 and NLRP3 expression compared with the L‐Js group (*p*  < 0.01), with effects comparable to those observed in the DOP group. For p38 MAPK, no significant difference was observed between the L‐Js group and the model group (*p*  > 0.05; not significant), whereas significant downregulation was detected in both the H‐Js and DOP groups (*p*  < 0.01). All protein expression levels were normalized to β‐actin, and the stability of β‐actin expression across groups was verified before normalization. Densitometric quantification was performed based on at least three independent biological replicates, and all analyses were conducted under blinded conditions.

## 4. Discussion

### 4.1. Neuroinflammation as a Central and Targetable Mechanism in AD‐Associated Cognitive Impairment

AD is clinically defined by progressive cognitive decline, with memory loss and impaired executive function as core features [30]. While hallmark pathologies such as Aβ accumulation and tau hyperphosphorylation are often emphasized, accumulating evidence suggests that neuroinflammation plays a major role in driving cognitive deficits, either independent of or in conjunction with classical proteinopathies in AD‐relevant contexts [31, 32]. Chronic activation of innate immune signaling pathways in the brain has been associated with synaptic dysfunction, impaired neuronal communication, and cognitive impairment in both preclinical models and human studies [33]. Activated microglia and astrocytes release a range of pro‐inflammatory mediators, including IL‐1β, TNF‐α, IL‐6, nitric oxide (NO), and ROS, which can disrupt neuronal homeostasis and synaptic plasticity. These inflammatory cascades are closely linked to the severity of cognitive deficits observed in experimental models and patient cohorts [34, 35]. Importantly, the interplay between neuroinflammatory signaling and neurodegenerative processes can create a self‐reinforcing loop, further exacerbating cognitive impairment without necessarily reflecting the full spectrum of AD pathology [36, 37].

Despite being a promising target, neuroinflammation has proven difficult to modulate clinically. NSAIDs, for example, have shown limited efficacy in AD, likely due to systemic side effects and their relatively narrow single‐target mechanisms. This underscores the need for multitarget therapeutic approaches capable of modulating neuroinflammatory networks, particularly those contributing to cognitive dysfunction. Such strategies may be more relevant to interventions aimed at preserving or improving cognitive function, independent of strict AD‐specific pathological endpoints.

### 4.2. *Z. jujuba*‐Mediated Modulation of Neuroinflammatory Networks in Cognitive Impairment Models Relevant to AD

Building upon the concept that sustained neuroinflammatory activation is a key contributor to cognitive dysfunction observed in AD‐relevant contexts (Section 4.1), increasing attention has focused on therapeutic agents capable of modulating key molecular mediators that link innate immune activation to synaptic dysfunction and memory decline. Among these, NF‐κB signaling, NLRP3 inflammasome activation, and prostaglandin (PG)‐related inflammatory cascades have been identified as important pathways through which chronic neuroinflammation may contribute to cognitive deficits.

In this context, TCM and food–medicine homologous agents offer a distinctive therapeutic paradigm, characterized by multitarget and network‐level regulation rather than single‐pathway inhibition. *Z. jujuba*, a widely consumed dietary fruit with a long history of medicinal use and an established safety profile, has attracted growing interest for its potential to attenuate neuroinflammatory processes implicated in cognitive dysfunction. Previous studies have demonstrated that *Z. jujuba*‐containing formulations or derived bioactive compounds exert anti‐inflammatory and neuroprotective effects, partly via modulation of TNF‐related signaling, HIF‐1 pathways, and PI3K/AKT cascades [38–40]. However, most of these studies relied on multi‐herb prescriptions or in vitro/in vivo models that do not directly isolate the effects of *Z. jujuba* as a single agent on cognitive function. To address this gap, we integrated network pharmacology, transcriptomic bioinformatics, and machine learning approaches to systematically delineate the molecular mechanisms by which *Z. jujuba* may potentially alleviate inflammation‐driven cognitive dysfunction. By integrating data from the GeneCards, GEO, and TCMSP databases, we identified 29 overlapping targets associated with AD‐relevant gene expression patterns and *Z. jujuba*‐related bioactive compounds. Functional enrichment analyses indicated that these targets were predominantly involved in cytokine production, inflammatory signaling pathways, receptor–ligand interactions, and endocytic processes—biological functions critically implicated in neuroinflammation‐mediated synaptic dysfunction and memory impairment [41, 42]. Collectively, these findings suggest that *Z. jujuba* may exert cognitive benefits through coordinated modulation of interconnected inflammatory and immune‐related networks, rather than through a single mediator. Such network‐level regulation is particularly relevant to cognitive impairment driven by sustained neuroinflammation, as modeled in scopolamine‐induced deficits, rather than serving as direct validation of AD‐specific pathological processes.

### 4.3. PTGS2 as a Key Inflammatory Node Linking NF‐κB Signaling, NLRP3 Inflammasome Activation, and Neuroinflammation‐Associated Cognitive Impairment

Building upon the concept that dysregulated neuroinflammation represents a central and targetable mechanism underlying cognitive impairment (Section 4.1), further prioritization of the 29 candidate genes was performed to identify key inflammatory regulators potentially modulated by *Z. jujuba*. By integrating three complementary machine learning algorithms (LASSO, RF, and SVM–RFE) with PPI network topological analysis, PTGS2 consistently emerged as a top‐ranked hub gene, highlighting its potential role as a key node within the neuroinflammatory network.

PTGS2 (COX‐2) is a well‐characterized inducible inflammatory enzyme that links upstream innate immune activation to downstream effector responses and has been implicated in neuroinflammation‐driven synaptic dysfunction and cognitive impairment [43, 44]. ROC curve analysis and diagnostic nomogram construction using AD‐relevant transcriptomic datasets demonstrated favorable discriminative performance (AUC > 0.7) and predictive consistency, supporting PTGS2 as a robust candidate mediator in neuroinflammation‐associated cognitive dysfunction. GSEA revealed that PTGS2‐associated genes were predominantly enriched in the NLR signaling pathway, consistent with KEGG enrichment results derived from the full target set. Molecular docking analyses further showed that 14 *Z. jujuba*‐derived bioactive compounds associated with PTGS2 exhibited stable binding affinities (average Δ*G* ≈ −8.5 kcal/mol). To explore potential CNS relevance, BBB permeability was assessed, and five representative compounds—beta‐carotene, stigmasterol, beta‐sitosterol, nuciferine, and berberine—were selected for docking visualization. These compounds displayed stable interactions within the PTGS2 active pocket via hydrogen bonding and hydrophobic contacts. However, it should be emphasized that the in vivo effects of *Z. jujuba* likely reflect the integrated activity of multiple constituents, including those with lower predicted BBB penetration, rather than being solely attributable to the five visualized compounds.

Mechanistically, PTGS2 is closely associated with key inflammatory processes, including NF‐κB activation, PG synthesis, and NLRP3 inflammasome signaling [45, 46]. NF‐κB activation upregulates PTGS2 transcription, increasing PGE2 production, which may amplify proinflammatory cytokine release and oxidative stress. This inflammatory environment facilitates NLRP3 inflammasome activation, resulting in IL‐1β maturation and downstream MAPK signaling, creating a positive feedback loop that sustains neuroinflammation and contributes to synaptic dysfunction [47, 48]. PTGS2’s chromosomal, neuroanatomical, and cellular localization further supports its pathological relevance: it is located on chromosome 1, predominantly expressed in the cerebral cortex, and mainly localized to vesicular compartments, which are important platforms for inflammatory signaling [49–52]. Experimental validation in a scopolamine‐induced cognitive impairment mouse model suggested that *Z. jujuba* treatment significantly improved spatial learning and memory performance in the MWM (*p*  < 0.01), preserved hippocampal neuronal architecture, and downregulated PTGS2, NLRP3, NF‐κB p65, p38 MAPK, and IL‐1β protein expression (*p*  < 0.01). Taken together, these findings support PTGS2 as a key node within the inflammatory network modulated by *Z. jujuba* in the context of neuroinflammation‐associated cognitive impairment, while acknowledging that additional targets and pathways may also contribute to the observed effects.

### 4.4. Translational Implications for Inflammation‐Driven Cognitive Impairment

Despite extensive evidence implicating neuroinflammation in AD, clinical translation of conventional anti‐inflammatory therapies has largely failed to improve cognitive outcomes. Most NSAIDs are designed to suppress acute inflammatory responses by targeting COX activity, yet they show minimal efficacy in preserving learning and memory in AD patients [53]. These observations suggest that cognitive impairment in AD arises not from a single inflammatory mediator but from sustained activation of interconnected neuroinflammatory networks that progressively compromise synaptic integrity and neuronal function. In this context, our study highlights a translational strategy focused on modulating inflammation‐driven cognitive dysfunction rather than global inflammation suppression. We demonstrate that *Z. jujuba* does not act as a conventional anti‐inflammatory agent; instead, it exerts coordinated regulatory effects on a PTGS2‐centered inflammatory network, encompassing NF‐κB signaling, NLRP3 inflammasome activation, MAPK pathways, and downstream pro‐inflammatory cytokine release [54]. These signaling axes are mechanistically linked to synaptic plasticity deficits, hippocampal dysfunction, and memory impairment, thereby directly connecting neuroinflammation to cognitive decline.

Importantly, the translational relevance of *Z. jujuba* lies not only in its multitarget, network‐level mechanism but also in its suitability for long‐term modulation of low‐grade chronic neuroinflammation that underlies progressive cognitive impairment. As a food–medicine homologous agent with an established safety profile, *Z. jujuba* is particularly well suited for early or preventive intervention aimed at delaying inflammation‐associated cognitive decline, a therapeutic window where conventional NSAIDs have consistently failed. The convergence of bioinformatics analyses, BBB‐informed molecular docking, and behavioral and molecular validation in a scopolamine‐induced cognitive impairment mouse model supports the potential feasibility of targeting PTGS2‐centered inflammatory networks as a translational approach to preserving cognitive function in AD‐relevant contexts.

### 4.5. Limitations and Future Directions

Despite the strengths of this study, several limitations should be acknowledged. First, although our integrative bioinformatics analyses, molecular docking, and in vivo validation collectively highlight PTGS2‐centered inflammatory regulation as a potential mechanism underlying the cognitive benefits of *Z. jujuba*, the neuroprotective effects of *Z. jujuba* have been reported in previous studies. Prior research using *Z. jujuba* seed extracts or isolated constituents in cellular and animal models demonstrated antioxidant, anti‐inflammatory, and cognition‐related benefits. Our study extends these findings by situating the effects of *Z. jujuba* within a systems‐level inflammatory network directly associated with cognitive dysfunction and by identifying PTGS2 as a central regulatory hub. An additional methodological consideration relates to WGCNA module selection. The MEturquoise module was chosen based on its strongest negative correlation with disease status (AD dataset) among all identified modules; however, this correlation did not reach conventional statistical significance (*r* = −0.31, *p* = 0.06). While this module served as an exploratory screening tool that subsequently yielded a robust, machine‐learning‐validated target (PTGS2), readers should interpret the initial co‐expression findings as hypothesis‐generating rather than confirmatory.

Second, the present study employed a whole‐fruit *Z. jujuba* suspension rather than isolated compounds, and no quantitative chemical profiling or chromatographic fingerprinting (e.g., HPLC or LC–MS/MS) was performed for the specific batch used. While the preparation follows standardized aqueous extraction procedures and the composition is consistent with published phytochemical data, the precise in vivo concentrations of individual constituents remain unknown. Although BBB permeability was evaluated post hoc to select representative compounds for docking visualization (logBB > 0.3), it was not used as a primary screening criterion during target identification. This approach allowed us to illustrate the potential CNS relevance of specific constituents (e.g., beta‐carotene, stigmasterol, beta‐sitosterol, nuciferine, and berberine) while acknowledging that the in vivo effects of *Z. jujuba* suspension likely reflect the combined actions of multiple compounds, including those with lower predicted BBB penetration or active metabolites. Future studies should integrate BBB permeability prospectively to enhance CNS‐targeted compound selection. Therefore, the observed cognitive and anti‐inflammatory effects likely reflect the integrated actions of multiple bioactive components rather than the effects of a single compound, and future work should incorporate chemical characterization and pharmacokinetic analyses to strengthen reproducibility and interpretability.

Third, although PTGS2 emerged as the most consistently ranked hub gene across machine learning, network topology, and experimental validation, cognitive impairment is inherently multifactorial, and focusing on a single inflammatory hub may underestimate the broader regulatory network through which *Z. jujuba* exerts its effects. Additional pathways, including cholinergic neurotransmission, oxidative stress, mitochondrial function, and synaptic plasticity, may contribute to its neuroprotective properties and were not directly assessed in this study. Future research using genetic or pharmacological manipulation of PTGS2 (e.g., knockdown or knockout models) will be valuable to clarify the specificity and necessity of PTGS2 signaling in mediating the observed effects. Finally, AD‐related transcriptomic datasets were used as representative sources to capture gene expression patterns associated with cognitive dysfunction, which is the primary clinical manifestation of AD. The scopolamine‐induced cognitive impairment model was employed as an in vivo system to experimentally validate the effects of *Z. jujuba* on learning and memory. While this model does not reproduce the full spectrum of AD‐specific pathological hallmarks such as Aβ accumulation or tau hyperphosphorylation, it is well‐established for evaluating interventions that modulate neuroinflammation‐driven cognitive deficits. Consequently, it provides a relatively translationally relevant platform to assess the potential efficacy of *Z. jujuba* in ameliorating inflammation‐associated cognitive impairment. Future studies using transgenic AD models, longer‐term interventions, and pharmacokinetic analyses—including BBB penetration assessments—will further strengthen the translational relevance of these findings.

## 5. Conclusion

This study demonstrates that *Z. jujuba* ameliorates neuroinflammation‐related cognitive dysfunction primarily through PTGS2‐centered modulation of inflammatory signaling pathways. By integrating computational analyses, machine learning, molecular docking of BBB‐permeable compounds, and in vivo behavioral and molecular validation, our findings provide mechanistic evidence that *Z. jujuba* acts as a safe, multitarget intervention for cognitive impairment driven by neuroinflammation. These results further highlight the therapeutic potential of food–medicine homologous agents in managing inflammation‐associated cognitive deficits.

## Author Contributions

Writing – original draft preparation: Qi Liu. Visualization: Binchuan Wang and Yihan Wang. Writing – review and editing: Jinyu Wang, Yi Liu, and Xuefeng Min. Funding acquisition: Mingwei Zhang.

## Acknowledgments

The authors thank all public databases.

## Funding

This study was supported by the Luzhou Science and Technology Plan Project (Grant 2022‐JYJ‐153) and the Natural Science Foundation of Southwest Medical University (Grant 2022QN114).

## Disclosure

All authors have read and agreed to the published version of the manuscript.

## Ethics Statement

Ethical Review Approval for Laboratory Animal Welfare at Southwest Medical University (Approval Number 20221026‐013).

## Consent

This study does not involve any human participants, and informed consent is therefore not applicable.

## Conflicts of Interest

The authors declare no conflicts of interest.

## Supporting Information

Additional supporting information can be found online in the Supporting Information section.

## Supporting information


**Supporting Information 1** Table S1. Detailed composition of *Z. jujuba* and corresponding machine learning–identified targets.

**Supporting Information 2** Detailed preparation procedure, representative chemical composition, and bioavailability considerations of *Z. jujuba*.

**Supporting Information 3** Figure S1. All quantitative analyses were performed in a blinded manner to minimize bias; representative full‐length blots are provided.

## Data Availability

All data generated or analyzed during this study are included in this published article and Supporting Information section.

## References

[1] 2023 Alzheimer’s Disease Facts and Figures, Alzheimer’s Dement. (2023) 19, no. 4, 1598–1695, 10.1002/alz.13016. 36918389

[2] Scheltens P. , De Strooper B. , and Kivipelto M. , et al.Alzheimer’s Disease, The Lancet. (2021) 397, no. 10284, 1577–1590, 10.1016/S0140-6736(20)32205-4. PMC8354300 33667416

[3] Khan S. , Barve K. H. , and Kumar M. S. , Recent Advancements in Pathogenesis, Diagnostics and Treatment of Alzheimer’s Disease, Current Neuropharmacology. (2020) 18, no. 11, 1106–1125, 10.2174/1570159X18666200528142429. 32484110 PMC7709159

[4] Si Z.-Z. , Zou C.-J. , and Mei X. , et al.Targeting Neuroinflammation in Alzheimer’s Disease: From Mechanisms to Clinical Applications, Neural Regeneration Research. (2023) 18, no. 4, 708–715, 10.4103/1673-5374.353484. 36204826 PMC9700083

[5] Liu Q. , Wang Y. , and Wang B. , et al.Pinostrobin Attenuates Microglia-Mediated Neuroinflammation After Subarachnoid Hemorrhage Through Modulation of the MYC–CTSL Signaling Axis, Naunyn-Schmiedeberg’s Archives of Pharmacology. (2026) 10.1007/s00210-026-05141-y. 41807810

[6] Chou V. , Pearse R. V.II, and Aylward A. J. , et al.INPP5D Regulates Inflammasome Activation in Human Microglia, Nature Communications. (2023) 14, no. 1, 10.1038/s41467-023-42819-w, 7552. PMC10684891 38016942

[7] Abdulkhaliq A. A. , Kim B. , and Almoghrabi Y. M. , et al.Amyloid-β and Tau in Alzheimer’s Disease: Pathogenesis, Mechanisms, and Interplay, Cell Death & Disease. (2026) 17, no. 1, 10.1038/s41419-025-08186-8, 21. 41513640 PMC12789470

[8] Zhang Y. , Qu S. Q. , and Yang Y. M. , et al.The Traditional Records and Application of *Ziziphus jujuba* , Traditional Chinese Medicine. (2021) 10, no. 5, 716–727, 10.12677/TCM.2021.105099.

[9] Wang J. , Analysis of Modern Research on Jujube Based on Literature Mining, Explor Rational Drug Use China. (2021) 18, no. 9, 20–23.

[10] Sobhani Z. , Nikoofal-Sahlabadi S. , Amiri M. S. , Ramezani M. , Emami S. A. , and Sahebkar A. , Therapeutic Effects of *Ziziphus jujuba* Mill. Fruit in Traditional and Modern Medicine: A Review, Medicinal Chemistry. (2020) 16, no. 8, 1069–1088, 10.2174/1573406415666191031143553. 31670624

[11] Mohebbati R. , Bavarsad K. , Rahimi M. , Rakhshandeh H. , Rad A. K. , and Shafei M. N. , Protective Effects of Long-Term Administration of *Ziziphus jujuba* Fruit Extract on Cardiovascular Responses in L-NAME Hypertensive Rats, Avicenna Journal of Phytomedicine. (2018) 8, no. 2, 143–151. 29632845 PMC5885328

[12] Alsayari A. and Wahab S. , Genus Ziziphus for the Treatment of Chronic Inflammatory Diseases, Saudi Journal of Biological Sciences. (2021) 28, no. 12, 6897–6914, 10.1016/j.sjbs.2021.07.076. 34866990 PMC8626254

[13] Cai W. , Zhuang H. , and Wang X. , et al.Functional Nutrients and Jujube-Based Processed Products in *Ziziphus jujuba* , Molecules. (2024) 29, no. 14, 10.3390/molecules29143437, 3437. 39065014 PMC11279998

[14] Xue X. , Zhao A. , and Wang Y. , et al.Composition and Content of Phenolic Acids and Flavonoids Among the Different Varieties, Development Stages, and Tissues of Chinese Jujube (*Ziziphus jujuba* Mill.), PLoS ONE. (2021) 16, no. 10, 10.1371/journal.pone.0254058, e0254058. 34648512 PMC8516285

[15] Jia F. , Wang B. , Ma H. , Bai C. , and Zhang Y. , Research Progress on Extraction, Separation, Structure, and Biological Activities of Polysaccharides From Jujube Fruit (*Ziziphus jujuba* Mill.): A Review, Frontiers in Chemistry. (2025) 13, 10.3389/fchem.2025.1581947, 1581947. 40308264 PMC12041218

[16] Shen D. , Wu C. , and Fan G. , et al.Jujube Peel Polyphenols Synergistically Inhibit Lipopolysaccharide-Induced Inflammation Through Multiple Signaling Pathways in RAW. 264.7 Cells, Food and Chemical Toxicology. (2022) 164, 10.1016/j.fct.2022.113062, 113062. 35460827

[17] Liu C. , Wang F. , and Zhang R. , An Acidic Polysaccharide With Anti-Inflammatory Effects From Blackened Jujube: Conformation and Rheological Properties, Foods. (2022) 11, no. 16, 10.3390/foods11162488, 2488. 36010488 PMC9407416

[18] Kim Y. , Oh J. , Jang C. H. , Lim J. S. , Lee J. S. , and Kim J.-S. , In Vivo Anti-Inflammatory Potential of Viscozyme®–Treated Jujube Fruit, Foods. (2020) 9, no. 8, 10.3390/foods9081033, 1033. 32752184 PMC7466189

[19] Huang W. , Wang Y. , Jiang X. , Sun Y. , Zhao Z. , and Li S. , Protective Effect of Flavonoids From *Ziziphus jujuba* cv. Jinsixiaozao Against Acetaminophen-Induced Liver Injury by Inhibiting Oxidative Stress and Inflammation in Mice, Molecules. (2017) 22, no. 10, 10.3390/molecules22101781, 2-s2.0-85032916425, 1781. 29053632 PMC6151471

[20] Zhu D. , Zhu Y. , and Tan H. , et al.Effects of Jujube (*Ziziphus jujuba* Mill.) Fruit Extracts on Oxidative Stress: A Systematic Review and Meta-Analysis of Rodent Studies, Food Science & Nutrition. (2024) 12, no. 8, 5312–5328, 10.1002/fsn3.4234. 39139963 PMC11317725

[21] Jain B. , Raj U. , and Varadwaj P. K. , Drug Target Interplay: A Network-Based Analysis of Human Diseases and the Drug Targets, Current Topics in Medicinal Chemistry. (2018) 18, no. 13, 1053–1061, 10.2174/1568026618666180719160922, 2-s2.0-85054979870. 30027850

[22] Li T. , Li W. , Guo X. , Tan T. , Xiang C. , and Ouyang Z. , Unraveling the Potential Mechanisms of the Anti-Osteoporotic Effects of the *Achyranthes bidentata*-*Dipsacus asper* Herb Pair: A Network Pharmacology and Experimental Study, Frontiers in Pharmacology. (2023) 14, 10.3389/fphar.2023.1242194, 1242194. 37849727 PMC10577322

[23] Yan M. , Pan D. , and Chen L. , et al.Role of Intestinal SCFAs Homeostasis in the Hepatoprotective Effect of *Clostridium butyricum* in T2DM, NPJ Biofilms and Microbiomes. (2025) 11, no. 1, 10.1038/s41522-025-00824-5, 206. 41219207 PMC12606137

[24] Miravalles C. , Cannon D. M. , and Hallahan B. , The Effect of Scopolamine on Memory and Attention: A Systematic Review and Meta-Analysis, European Psychiatry. (2025) 68, no. 1, 10.1192/j.eurpsy.2025.2446, e50. 40197394 PMC12041729

[25] Zhang J. , Wang J. , and Zhou G. S. , et al.Studies of the Anti-Amnesic Effects and Mechanisms of Single and Combined Use of Donepezil and Ginkgo Ketoester Tablet on Scopolamine-Induced Memory Impairment in Mice, Oxidative Medicine and Cellular Longevity. (2019) 2019, 10.1155/2019/8636835, 2-s2.0-85062555188, 8636835. 30911351 PMC6398023

[26] Huang J.-H. , Huang X. , and Chen Z. , et al.Equivalent Dose Conversion Between Animals and Between Animals and Humans in Pharmacological Tests, Chinese Clinical Pharmacology and Therapeutics. (2004) 9, no. 9, 1069–1072.

[27] Kim M. J. , Jung J. E. , Lee S. , Cho E. J. , and Kim H. Y. , Effects of the Fermented *Zizyphus jujuba* in the Amyloid β _25−35_ -Induced Alzheimer’s Disease Mouse Model, Nutrition Research and Practice. (2021) 15, no. 2, 173–186, 10.4162/nrp.2021.15.2.173. 33841722 PMC8007403

[28] Kilkenny C. , Browne W. J. , Cuthill I. C. , Emerson M. , and Altman D. G. , Improving Bioscience Research Reporting: The ARRIVE Guidelines for Reporting Animal Research, PLoS Biology. (2010) 8, no. 6, 10.1371/journal.pbio.1000412, 2-s2.0-85041813459, e1000412. 20613859 PMC2893951

[29] Wang X.-C. , Wu G.-L. , and Cai H.-Y. , et al.Jiao-Tai-Wan Improves Cognitive Impairment by Regulating Nrf2/ARE/HO-1 Signaling Pathway in APP/PS1 Mice, Neurochemical Research. (2025) 50, no. 5, 10.1007/s11064-025-04515-7, 268. 40824405

[30] Kobayashi G. , Hirata K. , Ono M. , Kasuga K. , and Takado Y. , The 2024 NIA-AA Biological Definition of Alzheimer’s Disease: Linking Biomarkers to Clinical Practice, Frontiers in Dementia. (2026) 5, 10.3389/frdem.2026.1736297, 1736297. 41756762 PMC12932163

[31] Bayraktaroglu I. , Ortí-Casañ N. , Van Dam D. , De Deyn P. P. , and Eisel U. L. M. , Systemic Inflammation as a Central Player in the Initiation and Development of Alzheimer’s Disease, Immunity & Ageing. (2025) 22, no. 1, 10.1186/s12979-025-00529-5, 33. 40841660 PMC12369153

[32] Gao C. , Jiang J. , Tan Y. , and Chen S. , Microglia in Neurodegenerative Diseases: Mechanism and Potential Therapeutic Targets, Signal Transduction and Targeted Therapy. (2023) 8, no. 1, 10.1038/s41392-023-01588-0, 359. 37735487 PMC10514343

[33] Rao J. S. , Kellom M. , Kim H.-W. , Rapoport S. I. , and Reese E. A. , Neuroinflammation and Synaptic Loss, Neurochemical Research. (2012) 37, no. 5, 903–910, 10.1007/s11064-012-0708-2, 2-s2.0-84862835163. 22311128 PMC3478877

[34] Li J.-M. , Hu T. , and Zhou X.-N. , et al.The Involvement of NLRP3 Inflammasome in CUMS-Induced AD-Like Pathological Changes and Related Cognitive Decline in Mice, Journal of Neuroinflammation. (2023) 20, no. 1, 10.1186/s12974-023-02791-0, 112. 37165444 PMC10173607

[35] Bathini P. , Dupanloup I. , and Zenaro E. , et al.Systemic Inflammation Causes Microglial Dysfunction With a Vascular AD Phenotype, Brain, Behavior, & Immunity - Health. (2023) 28, 10.1016/j.bbih.2022.100568, 100568. PMC9871075 36704658

[36] Chen Y. and Yu Y. , Tau and Neuroinflammation in Alzheimer’s Disease: Interplay Mechanisms and Clinical Translation, Journal of Neuroinflammation. (2023) 20, no. 1, 10.1186/s12974-023-02853-3, 165. 37452321 PMC10349496

[37] Kandimalla M. , Lim S. , and Jacobson D. N. , et al.Neuroinflammation Demonstrated by ^11^C-ER176 PET With Amyloid and Tau Pathology, Alzheimer’s & Dementia. (2026) 22, no. 1, 10.1002/alz.71027, e71027. PMC12828788 41574776

[38] Hao S. , Du Y. , and Lu S. , et al.Exploring the Mechanism of Action of Ganmai Dazao Tang in the Treatment of Cardiac Neurosis Based on Network Pharmacology Combined With Molecular Docking, Journal in Neuropharmacology. (2024) 14, no. 3, 36–46, 10.3969/j.issn.2095-1396.2024.03.005.

[39] Wang X. , Xie J. , and Jiang L. , Effects of Ganmai Dazao Tang Plus on Cognitive Function, Neurotransmitters, and Inflammatory Mediators in Depressed Patients After Stroke, Chinese Medical Information. (2023) 40, no. 6, 66–70, 10.19656/j.cnki.1002-2406.20230611.

[40] Chen C.-H. , Hsu P.-C. , and Hsu S.-W. , et al.Protective Effects of Jujubosides on 6-OHDA-Induced Neurotoxicity in SH-SY5Y and SK-N-SH Cells, Molecules. (2022) 27, no. 13, 10.3390/molecules27134106, 4106. 35807356 PMC9268520

[41] Yang G. , Xu X. , Gao W. , Wang X. , Zhao Y. , and Xu Y. , Microglia-Orchestrated Neuroinflammation and Synaptic Remodeling: Roles of Pro-Inflammatory Cytokines and Receptors in Neurodegeneration, Frontiers in Cellular Neuroscience. (2025) 19, 10.3389/fncel.2025.1700692, 1700692. 41292555 PMC12641016

[42] Bourgognon J.-M. and Cavanagh J. , The Role of Cytokines in Modulating Learning and Memory and Brain Plasticity, Brain and Neuroscience Advances. (2020) 4, 1–13, 10.1177/2398212820979802. PMC7750764 33415308

[43] Li X. , Li X. , and Zhang Q. , et al.Prostaglandin Endoperoxide Synthase 2 Regulates Neuroinflammation to Mediate Postoperative Cognitive Dysfunction in Mice, Scientific Reports. (2025) 15, no. 1, 10.1038/s41598-025-01121-z, 17355. 40389478 PMC12089394

[44] Moussa N. and Dayoub N. , Exploring the Role of COX-2 in Alzheimer’s Disease: Potential Therapeutic Implications of COX-2 Inhibitors, Saudi Pharmaceutical Journal. (2023) 31, no. 9, 10.1016/j.jsps.2023.101729, 101729. 37638222 PMC10448476

[45] Jiang J. and Dingledine R. , Prostaglandin Receptor EP2 in the Crosshairs of Anti-Inflammation, Anti-Cancer, and Neuroprotection, Trends in Pharmacological Sciences. (2013) 34, no. 7, 413–423, 10.1016/j.tips.2013.05.003, 2-s2.0-84879785100. 23796953 PMC4031445

[46] Tajdari M. , Peyrovinasab A. , Bayanati M. , Rabbani M. I. M. , Abdolghaffari A. H. , and Zarghi A. , Dual COX-2/TNF-α Inhibitors as Promising Anti-Inflammatory and Cancer Chemopreventive Agents: A Review, Iranian Journal of Pharmaceutical Research. (2024) 23, no. 1, 10.5812/ijpr-151312, e151312. 39830670 PMC11742592

[47] Yang C. , He Y. , and Ren S. , et al.Hydrogen Attenuates Cognitive Impairment in Rat Models of Vascular Dementia by Inhibiting Oxidative Stress and NLRP3 Inflammasome Activation, Advanced Healthcare Materials. (2024) 13, no. 20, 10.1002/adhm.202400400, e2400400. 38769944

[48] Xu J. and Núñez G. , The NLRP3 Inflammasome: Activation and Regulation, Trends in Biochemical Sciences. (2023) 48, no. 4, 331–344, 10.1016/j.tibs.2022.10.002. 36336552 PMC10023278

[49] Davies G. , Lam M. , and Harris S. E. , et al.Study of 300,486 Individuals Identifies 148 independent Genetic Loci Influencing General Cognitive Function, Nature Communications. (2018) 9, no. 1, 10.1038/s41467-018-04362-x, 2-s2.0-85048027481, 2098. PMC5974083 29844566

[50] Paudel Y. N. , Shaikh M. F. , and Chakraborti A. , et al.HMGB1: A Common Biomarker and Potential Target for TBI, Neuroinflammation, Epilepsy, and Cognitive Dysfunction, Frontiers in Neuroscience. (2018) 12, 10.3389/fnins.2018.00628, 2-s2.0-85055120274, 628. 30271319 PMC6142787

[51] Yang C. , Liu G. , Chen X. , and Le W. , Cerebellum in Alzheimer’s Disease and Other Neurodegenerative Diseases: An Emerging Research Frontier, MedComm. (2024) 5, no. 7, 10.1002/mco2.638, e638. 39006764 PMC11245631

[52] Dai J. , Li J. M. , and Xin H. , et al.Research Progress on Extracellular Vesicles in Alzheimer’s Disease, Chinese Journal of Alzheimer’s Disease and Related Disorders. (2025) 8, no. 2, 138–143, 10.3969/j.issn.2096-5516.2025.02.011.

[53] Imbimbo B. P. , Solfrizzi V. , and Panza F. , Are NSAIDs Useful to Treat Alzheimer’s Disease or Mild Cognitive Impairment?, Frontiers in Aging Neuroscience. (2010) 2, 10.3389/fnagi.2010.00019, 2-s2.0-84899519009, 19. 20725517 PMC2912027

[54] Kwon H. , Jung I. H. , and Yi J. H. , et al.The Seed of *Zizyphus jujuba* var. *spinosa* Attenuates Alzheimer’s Disease-Associated Hippocampal Synaptic Deficits Through BDNF/TrkB Signaling, Biological & Pharmaceutical Bulletin. (2017) 40, no. 12, 2096–2104, 10.1248/bpb.b17-00378, 2-s2.0-85035803107. 29199234

