# Supplementary material for: Ziziphus jujuba Mill. Suspension Ameliorates Scopolamine‐Induced Cognitive Impairment via PTGS2‐Centered Neuroinflammatory Signaling
Source: Mediators Inflamm. 2026 May 7;2026:8871660. doi: 10.1155/mi/8871660 (PMC13150435; doi:10.1155/mi/8871660)
Supplement: Supplementary file 2 — Supporting Information 2 Detailed preparation procedure, representative chemical composition, and bioavailability considerations of Z. jujuba. [file MI-2026-8871660-s002.docx]

**Preparation, Composition, and Bioavailability Considerations of Ziziphus jujuba Suspension**

**1. Preparation of Ziziphus jujuba Suspension**

**The Ziziphus jujuba suspension used for animal experiments was prepared by the Chinese Medicine Pharmacy of the Affiliated Hospital of Southwest Medical University following standardized decoction procedures consistent with clinical practice [S1, S2]. The raw materials were obtained from a certified supplier (Sichuan Chinese Herbal Pieces Co., Ltd., China; batch no. 20230715-ZJJ), and botanical identification was performed in accordance with pharmacopoeial standards to ensure authenticity.**

**Quality control of the crude drug and prepared decoction pieces was conducted by a qualified testing center in strict accordance with the standards of the** Chinese Pharmacopoeia **(2015 edition),** Sichuan Provincial Standards for Chinese Medicinal Materials **(2010 edition), and** Processing Specifications for Chinese Herbal Pieces of Sichuan Province **(2015 edition). Comprehensive evaluations were performed, including macroscopic and microscopic identification, content determination, pesticide residue analysis, aflatoxin detection, heavy metal assessment, DNA authentication, sulfur dioxide residue testing, and microbiological examination.**

Briefly, authenticated dried fruits of Ziziphus jujuba Mill. were rinsed with distilled water and subjected to two consecutive hot-water extractions at a solid–liquid ratio of 1:15 (w/v). Each extraction was performed by decocting at 100 °C for 60 min. The two extracts were combined, filtered through sterile gauze to remove insoluble residues, and gently concentrated under reduced pressure. The final extract was homogenized to yield a stable brownish-red translucent suspension containing water-soluble phytochemicals and colloidal fractions. **The prepared suspension exhibited a uniform appearance without visible precipitation or phase separation during the storage period, indicating acceptable physical stability.**

The suspension was freshly prepared or stored at 4 °C for no longer than 72 h prior to use to ensure chemical stability. The administered doses (100 and 200 mg/kg/day) were calculated based on the equivalent crude drug content. All batches were prepared using the same raw material source and standardized extraction protocol to ensure consistency and reproducibility across experiments.

**2. Chemical Composition of Ziziphus jujuba**

Based on previous phytochemical studies and pharmacopoeial standards, Ziziphus jujuba contains multiple classes of bioactive constituents relevant to neuroinflammation and cognitive regulation, including:1.Flavonoids: quercetin, rutin, kaempferol derivatives;2.Polysaccharides: acidic and neutral heteropolysaccharides with immunomodulatory activity;3.Triterpenoids: ursolic acid, oleanolic acid, and related saponins;4.Jujubosides: jujuboside A and B, known for neuroprotective and sedative effects;5.Phenolic acids and organic acids[S3-S6].

These constituents have been reported to exhibit anti-inflammatory, antioxidant, neuroprotective, and microglia-modulating activities, which are mechanistically relevant to neuroinflammation-driven cognitive impairment.Although quantitative chemical profiling (e.g., LC–MS/MS) was not performed in the present study, the composition of the suspension is consistent with previously reported profiles of water-extracted Z. jujuba preparations used in pharmacological research.

**3. Bioavailability and Food-Matrix Considerations**

As a typical “food–medicine homology” substance, Ziziphus jujuba is traditionally administered as a whole-extract preparation rather than isolated compounds. The aqueous suspension retains a complex food matrix composed of soluble fibers, polysaccharides, and colloidal particles, which may influence the absorption and metabolism of phytonutrients.Previous studies indicate that:1.Flavonoids such as quercetin and rutin exhibit moderate oral bioavailability, particularly when administered within a carbohydrate-rich matrix;2.Polysaccharides from Z. jujuba are poorly absorbed intact but exert biological effects through immune modulation, gut–brain axis regulation, and microglial signaling;3.Triterpenoids, although low in plasma concentration, display high target affinity and prolonged tissue retention[S7-S10].

While the precise physiological concentrations of individual constituents in plasma or brain tissue after Z. jujuba intake were not measured in this study, existing evidence supports the biological plausibility of central nervous system effects following oral administration, especially under chronic dosing conditions.

**4. Rationale for Using Whole Ziziphus jujuba Suspension**

Given the multifactorial pathogenesis of AD-like cognitive impairment, the use of a multi-component whole extract better reflects the holistic pharmacological characteristics of Ziziphus jujuba. This approach aligns with network pharmacology principles and allows simultaneous modulation of multiple inflammatory and neurodegenerative pathways, rather than relying on single-target interventions.

**Supplementary References**

[S1]Popstoyanova D, Gerasimova A, Gentscheva G, Nikolova S, Gavrilova A, Nikolova K. Ziziphus jujuba: Applications in the Pharmacy and Food Industry. *Plants (Basel)*. 2024;13(19):2724. Published 2024 Sep 29. doi:10.3390/plants13192724

[S2]Jia F, Wang B, Ma H, Bai C, Zhang Y. Research progress on extraction, separation, structure, and biological activities of polysaccharides from jujube fruit (Ziziphus jujuba Mill.): a review. *Front Chem.* 2025;13:1581947. Published 2025 Apr 16. doi:10.3389/fchem.2025.1581947

[S3]Cai W, Zhuang H, Wang X, et al. Functional Nutrients and Jujube-Based Processed Products in Ziziphus jujuba. *Molecules*. 2024;29(14):3437. Published 2024 Jul 22. doi:10.3390/molecules29143437

[S4]Xue X, Zhao A, Wang Y, et al. Composition and content of phenolic acids and flavonoids among the different varieties, development stages, and tissues of Chinese Jujube (Ziziphus jujuba Mill.). *PLoS One.* 2021;16(10):e0254058. Published 2021 Oct 14. doi:10.1371/journal.pone.0254058

[S5]Zhu D, Jiang N, Wang N, Zhao Y, Liu X. A Literature Review of the Pharmacological Effects of Jujube. *Foods*. 2024;13(2):193. Published 2024 Jan 6. doi:10.3390/foods13020193

[S6]Wu Y, Sun L, Ma Y, et al. Decoding multicomponent crosstalk: integrated pharmacodynamic‒pharmacokinetic network of sour jujube seed. *Chin Med*. 2025;20(1):195. Published 2025 Nov 20. doi:10.1186/s13020-025-01265-0

[S7]Liu L, Barber E, Kellow NJ, Williamson G. Improving quercetin bioavailability: A systematic review and meta-analysis of human intervention studies. *Food Chem*. 2025;477:143630. doi:10.1016/j.foodchem.2025.143630

[S8]Jiao L, Zou J, Ma H, Huang D, Li C. Regulatory effects of jujube (*Ziziphus jujuba*) polysaccharides on intestinal microbiota before and after α-galactosidase-mediated degradation. *Food Funct*. Published online January 2, 2026. doi:10.1039/d5fo04469c

[S9]J C Furtado NA, Pirson L, Edelberg H, et al. Pentacyclic Triterpene Bioavailability: An Overview of In Vitro and In Vivo Studies.*Molecules.* 2017;22(3):400. Published 2017 Mar 4. doi:10.3390/molecules22030400

[S10]Han X , Bai B , Zhou Q , et al. Dietary supplementation with polysaccharides from Ziziphus Jujuba cv. Pozao intervenes in immune response via regulating peripheral immunity and intestinal barrier function in cyclophosphamide-induced mice.*Food Funct.* 2020;11(7):5992-6006. doi:10.1039/d0fo00008f
