# Supplementary material for: Ziziphus jujuba Mill. Suspension Ameliorates Scopolamine‐Induced Cognitive Impairment via PTGS2‐Centered Neuroinflammatory Signaling
Source: Mediators Inflamm. 2026 May 7;2026:8871660. doi: 10.1155/mi/8871660 (PMC13150435; doi:10.1155/mi/8871660)

PTGS2 72kDa ß-actin 42kDa Control Model L-JS H-JS DOP

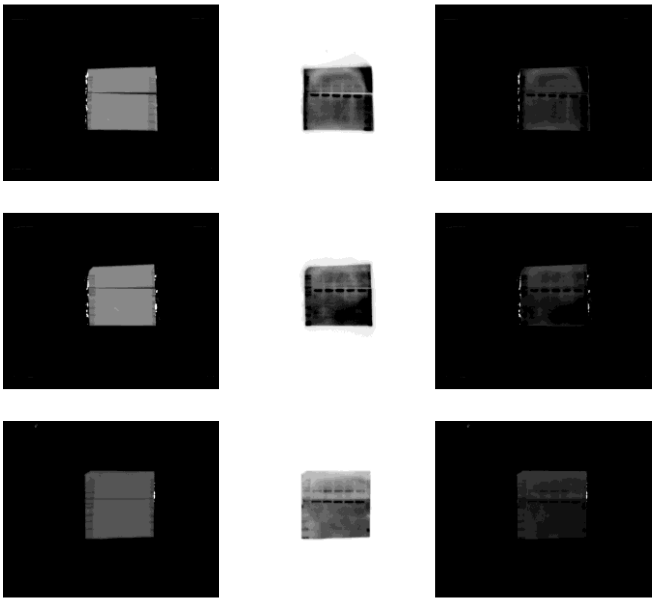

IL-1ß 17kDa ß-actin 42kDa Control Model L-JS H-JS DOP

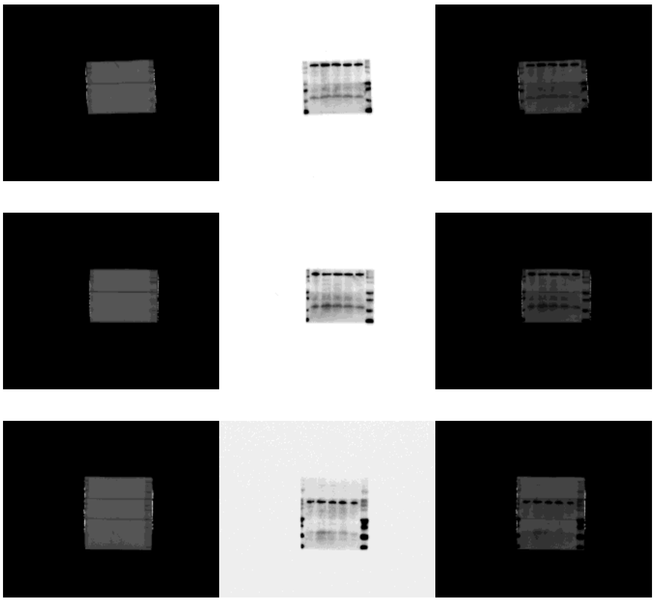

p38 MAPK 38kDa ß-actin 42kDa Control Model L-JS H-JS DOP

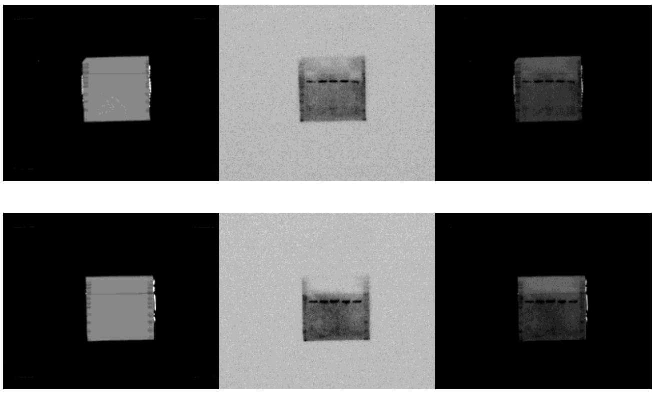

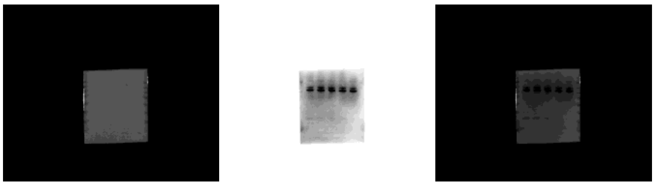

NF-κB p65 65kDa ß-actin 42kDa Control Model L-JS H-JS DOP

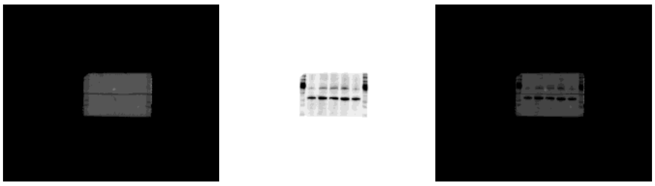

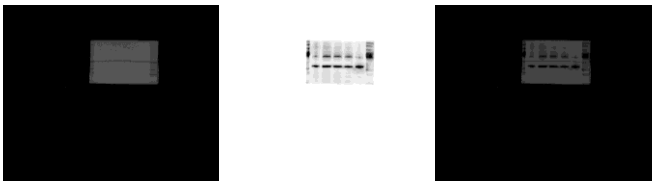

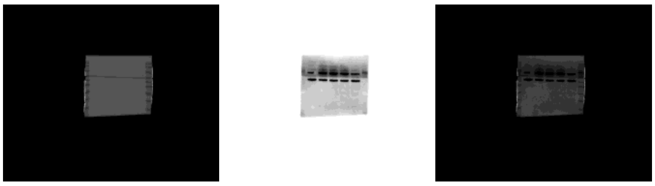

NLRP3 110kDa ß-actin 42kDa Control Model L-JS H-JS DOP

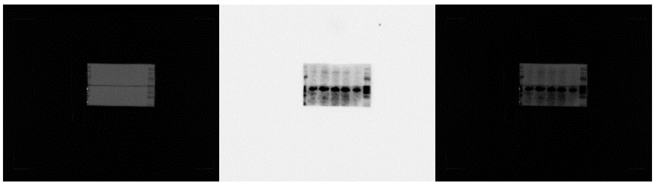

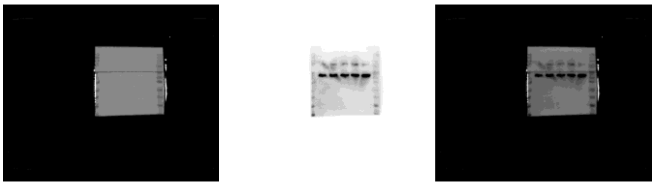

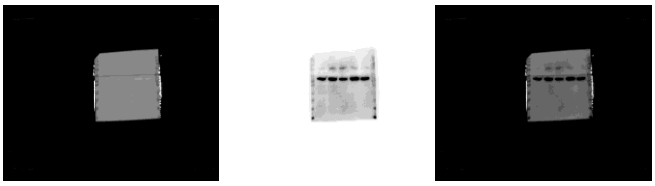

Supplement: Supplementary file 3 — Supporting Information 3 Figure S1. All quantitative analyses were performed in a blinded manner to minimize bias; representative full‐length blots are provided. [file MI-2026-8871660-s003.pdf]
